# Ebolavirus Is Internalized into Host Cells *via* Macropinocytosis in a Viral Glycoprotein-Dependent Manner

**DOI:** 10.1371/journal.ppat.1001121

**Published:** 2010-09-23

**Authors:** Asuka Nanbo, Masaki Imai, Shinji Watanabe, Takeshi Noda, Kei Takahashi, Gabriele Neumann, Peter Halfmann, Yoshihiro Kawaoka

**Affiliations:** 1 Influenza Research Institute, Department of Pathological Sciences, School of Veterinary Medicine, University of Wisconsin-Madison, Madison, Wisconsin, United States of America; 2 ERATO Infection-Induced Host Responses Project, Japan Science and Technology Agency, Saitama, Japan; 3 Division of Virology, Department of Microbiology and Immunology, Institute of Medical Science, University of Tokyo, Minato-ku, Tokyo, Japan; 4 International Research Center for Infectious Diseases, Institute of Medical Science, University of Tokyo, Minato-ku, Tokyo, Japan; Institut Pasteur, France

## Abstract

Ebolavirus (EBOV) is an enveloped, single-stranded, negative-sense RNA virus that causes severe hemorrhagic fever with mortality rates of up to 90% in humans and nonhuman primates. Previous studies suggest roles for clathrin- or caveolae-mediated endocytosis in EBOV entry; however, ebolavirus virions are long, filamentous particles that are larger than the plasma membrane invaginations that characterize clathrin- or caveolae-mediated endocytosis. The mechanism of EBOV entry remains, therefore, poorly understood. To better understand Ebolavirus entry, we carried out internalization studies with fluorescently labeled, biologically contained Ebolavirus and Ebolavirus-like particles (Ebola VLPs), both of which resemble authentic Ebolavirus in their morphology. We examined the mechanism of Ebolavirus internalization by real-time analysis of these fluorescently labeled Ebolavirus particles and found that their internalization was independent of clathrin- or caveolae-mediated endocytosis, but that they co-localized with sorting nexin (SNX) 5, a marker of macropinocytosis-specific endosomes (macropinosomes). Moreover, the internalization of Ebolavirus virions accelerated the uptake of a macropinocytosis-specific cargo, was associated with plasma membrane ruffling, and was dependent on cellular GTPases and kinases involved in macropinocytosis. A pseudotyped vesicular stomatitis virus possessing the Ebolavirus glycoprotein (GP) also co-localized with SNX5 and its internalization and infectivity were affected by macropinocytosis inhibitors. Taken together, our data suggest that Ebolavirus is internalized into cells by stimulating macropinocytosis in a GP-dependent manner. These findings provide new insights into the lifecycle of Ebolavirus and may aid in the development of therapeutics for Ebolavirus infection.

## Author Summary

Ebolavirus (EBOV) is an enveloped, single-stranded, negative-sense RNA virus that causes severe hemorrhagic fever with high mortality rates in humans and nonhuman primates. Previous studies suggest roles for clathrin- or caveolae-mediated endocytosis in EBOV entry; however, questions remain regarding the mechanism of EBOV entry. Here, we demonstrate that internalization of EBOV particles is independent of clathrin- or caveolae-mediated endocytosis. Specifically, we show that internalized EBOV particles co-localize with macropinocytosis-specific endosomes (macropinosomes) and that their entry is negatively affected by treatment with macropinocytosis inhibitors. Moreover, the internalization of Ebola virions accelerated the uptake of a macropinocytosis-specific cargo, was associated with plasma membrane ruffling, and was dependent on cellular GTPases and kinases involved in macropinocytosis. We further demonstrate that a pseudotyped vesicular stomatitis virus possessing the EBOV glycoprotein (GP) also co-localizes with macropinosomes and its internalization is similarly affected by macropinocytosis inhibitors. Our results indicate that EBOV uptake into cells involves the macropinocytic pathway and is GP-dependent. These findings provide new insights into the lifecycle of EBOV and may aid in the development of therapeutics for EBOV infection.

## Introduction

Viruses have evolved a variety of mechanisms to enter host cells [1], [2], [3], including clathrin- and caveolae-mediated endocytosis, phagocytosis, and macropinocytosis. The main route of endocytosis, mediated by clathrin, is characterized by the formation of clathrin-coated pits (CCP) of 85–110 nm in diameter that bud into the cytoplasm to form clathrin-coated vesicles. Influenza virus, vesicular stomatitis virus (VSV) and Semliki forest virus all enter their host cells *via* this pathway [4], [5], [6]. Although *Listeria monocytogenes* is larger than a CCP in diameter, it exploits non-classical clathrin-mediated endocytosis along with actin rearrangement to facilitate its infection [7], [8]. Caveolae are small vesicles of 50–80 nm in diameter enriched in caveolin, cholesterol, and sphingolipid, and have been implicated in simian virus 40 (SV40) entry [9]. Clathrin- and caveolae-mediated endocytosis requires large guanosine tryphosphatases (GTPase) dynamin 2 for vesicle scission [3].

Phagocytosis plays a role in the uptake of microorganisms, cell debris, and apoptotic cells [10]. It is initiated by the interaction of cell surface receptors, such as mannose receptors, Fc receptors and lectin receptors, with their ligands at the surface of the internalized particles. Particles are internalized through a dynamin 2- and actin-dependent mechanism [11] that results in the formation of phagosomes, large particles of >500 nm in diameter. Human herpes simplex virus and acanthamoeba polyphaga mimivirus are internalized through this mechanism [12], [13].

Macropinocytosis is characterized by actin-dependent membrane ruffling and, unlike phagocytosis, was thought to be independent of receptors or dynamin 2 [14], [15], [16], [17]. Macropinocytosis is constitutively activated in some immune cells, such as dendritic cells and macrophages [18], [19], [20]. In the other cell types, including epithelial cells and fibloblasts, macropinocytosis is initiated by growth factor stimulation [21], [22] or expression of ruffling kinases [23], [24], [25]. Macropinocytosis is also associated with the activation of Rho GTPases, such as Rac1 and Cdc42, which are responsible for triggering membrane ruffles by actin polymerization [26], [27], [28], [29]. Macropinocytosis is dependent on a series of kinases; a serine/threonine p21-activated kinase 1 (Pak1) is activated by Rac1 or Cdc42 and is essential for the regulation of cytoskeleton dynamics [24], [30]. In addition Pak1 plays a role in macropinosome closure by activating carboxy-terminal-binding protein-1/brefeldin A-ADP ribosylated substrate (CtBP-1/BARS) [30]. Phosphatidylinositol-3-kinase (PI3K) and its effectors are responsible for ruffling and macropinocytosis [23], [31]. Protein kinase C (PKC) is activated by a receptor tyrosine kinase or PI3K and also promotes plasma membrane ruffling and macropinocytosis [23]. Membrane ruffling is associated with the formation of macropinocytosis-specific endosomes, macropinosomes, of approximately 0.5–10 µm in diameter [32]. Human adenovirus type 3 (Ad3) [33], vaccinia virus [26], Kaposi's Sarcoma Associated Herpesvirus [34], and Nipah virus [35] enter cells *via* macropinocytosis. Human immunodeficiency virus (HIV) [36], [37] and Ad2/5 [38] may also trigger this pathway.

Ebolavirus (EBOV) is an enveloped, single-stranded, negative-sense RNA virus that belongs to the family *Filoviridae*. In humans and nonhuman primates, it causes severe hemorrhagic fever with mortality rates of up to 90%. Ebolavirus virions are long, filamentous particles of varied length (typically, 1–2 µm) and a diameter of 80–100 nm. EBOV infects a wide range of host cells [39], suggesting that its entry into target cells is mediated by the binding of its surface glycoprotein (GP) to a widely expressed and highly conserved receptor, or by GP binding to different host receptors. Several cellular proteins have been reported as EBOV receptors or co-receptors, including folate receptor-α (FR-α) [40], several lectins [41], [42], [43], [44], [45], [46], and integrin ß1 [47]. In addition, EBOV entry is facilitated by members of the Tyro3 protein kinase family [48], [49].

The mechanism of EBOV cell entry is currently poorly understood. EBOV is likely internalized by an endocytic pathway, since its entry is dependent upon low pH [50], [51] and the endocytic enzymes cathepsin B and L [52], [53], [54], [55], [56]. Several studies suggest that EBOV internalization depends on cholesterol, a major component of caveolae and lipid-rafts [50], [57], [58]. Another study suggests a role for clathrin-mediated endocytosis in wild-type EBOV and retrovirus psendotyped with EBOV GP entry [59], [60]. These discrepancies may reflect differences in the experimental systems and/or conditions used. Most studies have been carried out with retroviruses or vesicular stomatitis virus (VSV) pseudotyped with EBOV GP [52], [53], [54], [56], [58], [61]. These pseudotyped systems have limitations because the morphology of the virions differs significantly from that of authentic Ebola virions (spherical for retrovirus or VSV-pseudotyped virions versus filamentous for authentic Ebola virions).

To better understand EBOV entry, we conducted internalization studies with fluorescently labeled, biologically contained EBOV [62], and Ebolavirus-like particles (Ebola VLPs), both of which resemble authentic EBOV in their morphology [62], [63], [64], [65]. Our results suggest that EBOV uptake into cells involves the macropinocytic pathway and is GP-dependent**.**

## Results

### Internalization of fluorescently labeled Ebola virions and Ebolavirus-like particles (Ebola VLPs)

To assess the mechanism of EBOV entry, we established a real-time monitoring system for fluorescently labeled, biologically contained Ebola virions [62], and fluorescently labeled Ebola VLPs [63], [64], [65]. The biologically contained EBOV (EbolaΔVP30) lacks the gene for the viral transcriptional co-activator VP30 and can only replicate in VP30-expressing cells [62]. EbolaΔVP30 resembles authentic EBOV [62] and thus provides an ideal system to study EBOV entry. Likewise, co-expression of the EBOV GP glycoprotein and the VP40 matrix protein yields virus-like particles (VLPs) with filamentous architecture [63], [64], [65]. Since co-expression of the EBOV nucleoprotein (NP) increases the efficiency of VLP generation [66], we generated VLPs by co-expressing GP, VP40, and NP. To establish a real-time monitoring system for EBOV cell entry, EbolaΔVP30 virions and Ebola VLPs were generated and purified as described in the Material and Methods, and labeled with a lipophilic tracer, 1,1′-dioctadecyl-3,3,3′,3′-tetramethylindocarbocyanine perchlorate (DiI), which is incorporated into the envelope of the virions [67], [68], [69]. The infectivity of DiI-labeled EbolaΔVP30 was equivalent to that of unlabeled virions as measured by plaque assays (data not shown), demonstrating that DiI labeling did not interfere with virion binding and infectivity.

We synchronized the adsorption of DiI-labeled EbolaΔVP30 and Ebola VLPs to African green monkey kidney epithelial (Vero) cells, which support EBOV replication, for 30 min on ice. We assessed the effect of low temperature incubation on the internalization of the DiI-virions by incubation on ice, room temperature, or 37°C in parallel, followed by a temperature shift to 37°C and found that there were no appreciably differences in the total numbers of internalized virions across these conditions, suggesting that incubation of cells and virions on ice had a limited effect on the subsequent viral internalization (Figure S1).

After adsorption, we shifted the temperature to 37°C and visualized the labeled particles by using confocal laser scanning microscope at various times. DiI-labeled EbolaΔVP30 and Ebola VLPs were visualized as red particles of various sizes (red, Figure S2A), indicating that viral particles of various lengths had been produced, an observation that we confirmed by electron microscope (Figure S3). Both DiI-labeled EbolaΔVP30 and Ebola VLPs were internalized efficiently, migrated immediately after the temperature shift, and eventually trafficked to intracellular compartments (Figure S2A and B, left panels, Video S1). As a control, we tested VLPs that lacked GP [Ebola VLPs (-GP)]. These particles bound to the cells with low efficiency and remained stationary even after long-term incubation at 37°C (Figure S2A and B, right panels, Video S2), confirming the requirement of GP for binding and internalization of EBOV.

### Role of clathrin-mediated endocytosis in EBOV entry

**Figure 1:**
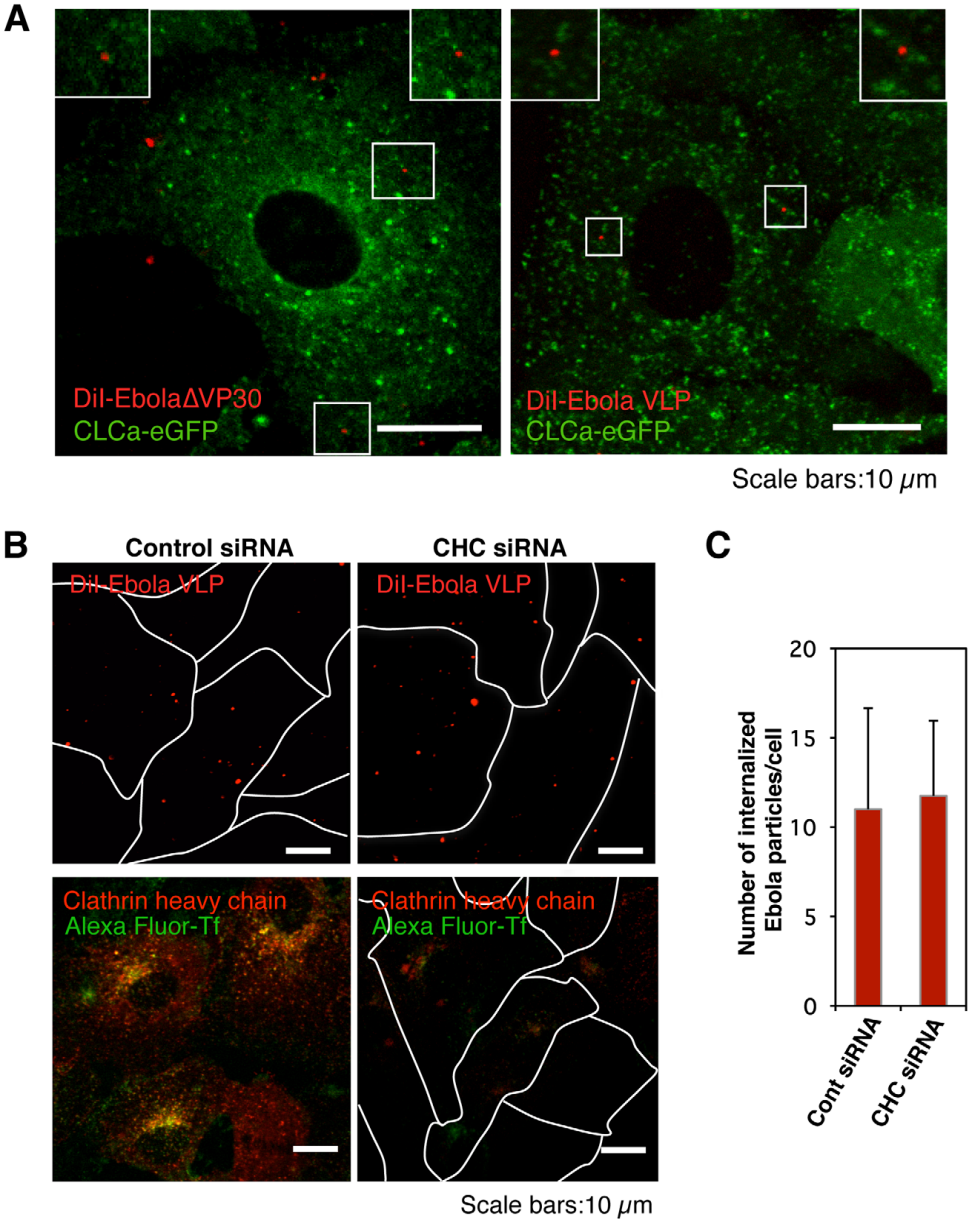
Internalization of DiI-labeled Ebola virions is independent of the clathrin -mediated endocytic pathway. (A) DiI-labeled Ebola virions (red) do not co-localize with eGFP-labeled CCPs. DiI-EbolaΔVP30 virions (left panel) or DiI-Ebola VLPs (right panel) were adsorbed to Vero cells expressing CLCa-eGFP for 30 min on ice. Cells were then incubated for 15 min at 37°C and the co-localization of DiI-labeled viral particles with eGFP-labeled CCPs was analyzed by using confocal microscope. Insets show enlargements of the boxed areas. Scale bars, 10 µm. (B) Effect of clathrin-heavy chain down-regulation on the internalization of DiI-labeled Ebola virions. Vero cells were transfected with control siRNA (left panels) or CHC siRNA (right panels) to down-regulate CHC expression. The efficiency of CHC down-regulation was analyzed by immunofluorescent staining 48 h post-transfection (red; lower panels); the effect of siRNA on Alexa Fluor 633-Tf is apparent (green; lower panels). Labeled Ebola VLPs were adsorbed to the siRNA-transfected cells for 30 min on ice 48 h post-transfection. After incubation for 2 h at 37°C, surface-bound virions were removed by the addition of trypsin for 5 min at 37°C and the internalization of Ebola VLPs was analyzed by using confocal laser scanning microscope (upper panels). Outlines of individual cells were drawn. Scale bars, 10 µm. (C) Quantitative analysis of the internalization of DiI-labeled Ebola virions in siRNA-transfected Vero cells. The number of DiI-virions in 10 individual siRNA-transfected cells was measured. Each experiment was performed in triplicate and the results are presented as the mean ± SD.

Previous studies suggested that EBOV enters cells *via* clathrin-mediated endocytosis [50], [59]. The typical architecture of Ebola virions (length 1–2 µm and diameter 80–100 nm) is larger than the diameter of clathrin-coated pits (85–110 nm). However, *Listeria monocytogenes* is internalized into cells *via* non-classical clathrin-mediated endocytosis [7], [8], Therefore, we visualized clathrin-coated pits (CCPs) *via* the expression of clathrin light chain a (CLCa) fused to enhanced green fluorescent protein (eGFP) to assess the significance of this pathway for EBOV internalization. The functional integrity of clathrin is not compromised by fusion to eGFP and the expressed fusion protein forms CCPs with endogenous CLCa [70], [71]. We did not detect co-localization of eGFP-labeled CLCa (CLCa-eGFP) with DiI-labeled EbolaΔVP30 virions (Figure 1A, left panel and Video S3) or Ebola VLPs (Figure 1A, right panel) at 15 min or 60 min after the temperature shift, whereas fluorescence-labeled Transferrin (Tf), a specific ligand of the clathrin-mediated pathway, was co-localized with eGFP-CLCa (Figure S4, left panel). These results suggest that clathrin-mediated endocytosis may not be critical for EBOV entry.

To further assess the role of clathrin-dependent endocytosis in EBOV entry, we down-regulated endogenous clathrin heavy chain (CHC) with small interfering RNAs (siRNA) and assessed the effect of CHC down-regulation on the internalization of Ebola virions. Down-regulation of CHC expression (red) was confirmed by immunofluorescent staining in Vero cells (Figure 1B, lower right panel). To remove the surface-bound uninternalized virions, we treated the cells with trypsin 2 h post-temperature shift (Figure S5). The uptake of Alexa Fluor-Tf was abrogated in CHC siRNA-treated cells, indicating that the clathrin-mediated endocytosis was blocked in these cells (Figure 1B, lower right panel). However, internalization of Ebola VLPs was not blocked by down-regulation of CHC (Figure 1B, upper right panel and Figure 1C), further suggesting that clathrin-mediated endocytosis is not critical for EBOV entry.

### Role of caveolin-mediated endocytosis in EBOV entry

**Figure 2:**
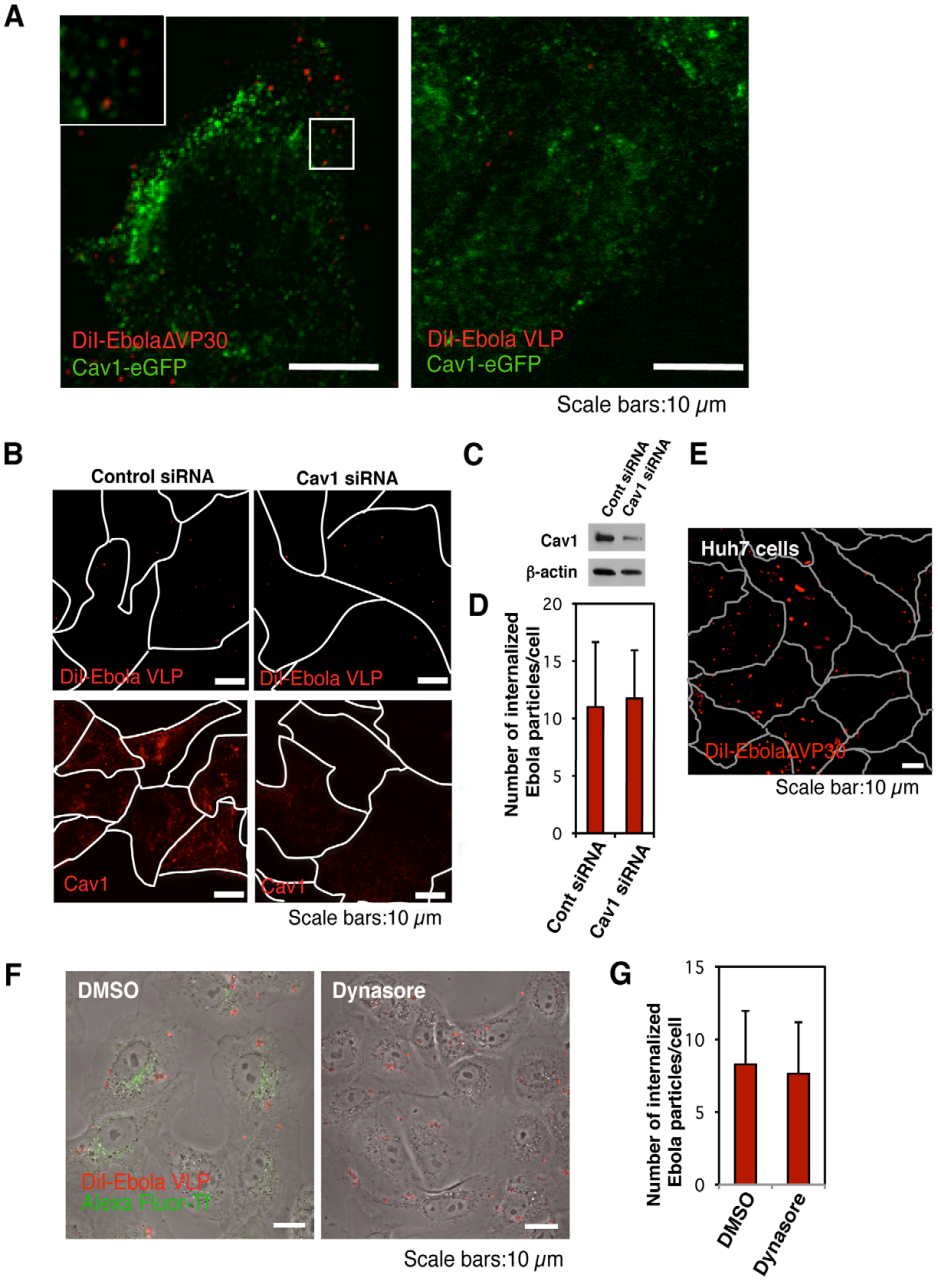
Internalization of DiI-labeled EBOV particles is independent of the caveolae-mediated endocytic pathway. (A) DiI-labeled EBOV particles do not co-localize with eGFP-labeled caveolae. DiI-EbolaΔVP30 virions (left panel) or DiI-Ebola VLPs (right panel) were adsorbed to Cav1-eGFP-expressing Vero cells for 30 min on ice. The cells were then incubated for 15 min at 37°C and the co-localization of DiI-labeled viral particles with eGFP-labeled caveolae was analyzed by using confocal laser scanning microscope. Insets show enlargements of the boxed areas. Scale bars, 10 µm. (B) Effect of Cav1 down-regulation on the internalization of DiI-labeled Ebola virions. Vero cells were transfected with control siRNA (left panels) or siRNA to down-regulate Cav1 expression (right panels). The efficiency of Cav1 down-regulation was analyzed by use of immunofluorescent staining 48 h post-transfection (lower panels) and western blot analysis (C). Labeled Ebola VLPs were adsorbed to the siRNA-transfected cells for 30 min on ice 48 h post-transfection. After incubation for 2 h at 37°C, surface-bound virions were removed by the addition of trypsin for 5 min at 37°C and the internalization of Ebola VLPs was analyzed by using confocal laser scanning microscope (upper panels). Outlines of individual cells were drawn. Scale bars, 10 µm. (D) Quantitative analysis of the internalization of DiI-labeled Ebola virions in siRNA-transfected Vero cells. The internalized DiI-virions were analyzed in 10 individual siRNA-transfected cells. Each experiment was performed in triplicate and the results are presented as the mean ± SD. (E) Internalization of DiI-labeled Ebola virions in cells lacking Cav1. DiI-labeled EbolaΔVP30 virions were adsorbed to Cav1-deficient Huh7 cells for 30 min on ice. The internalization of DiI-EbolaΔVP30 virions was analyzed 2 h after the temperature shift to 37°C. Outlines of individual cells were drawn. Scale bar, 10 µm. (F) Effect of dynasore on the internalization of DiI-labeled Ebola virions. Vero cells were treated with DMSO (left panel) or dynasore (right panel) for 30 min at 37°C. Labeled Ebola VLPs were adsorbed to the cells for 30 min on ice and incubated for 2 h at 37°C in the presence of DMSO or dynasore. Surface-bound virions were removed by trypsin and the internalization of DiI-virions was analyzed by using confocal laser scanning microscope. Dynasore treatment interfered with the internalization of Alexa Fluor 633-Tf (green in right panel), attesting to its functionality. Scale bars, 10 µm. (G) Quantitative analysis of the internalization of DiI-labeled Ebola virions in dynasore-treated Vero cells. The internalized DiI-virions were analyzed in 10 individual DMSO- or dynasore-treated cells. Each experiment was performed in triplicate and the results are presented as the mean ± SD.

Previous studies also indicated a role for caveolin-mediated endocytosis in EBOV internalization [50], [59]. Using a similar strategy to that described above, we assessed the co-localization of eGFP-fused caveolin 1 (Cav1-eGFP), which does not impair the internalization of caveolae [9], [72], with DiI-labeled EbolaΔVP30 (Figure 2A, left panel, Video S4) and Ebola VLPs (Figure 2A, right panel). We did not observe efficient co-localization of labeled Ebola virions with Cav1, indicating that caveolaes may not play a critical role in EBOV entry. Alexa Fluor-Chorela toxin B subunit (CtxB), which is internalized *via* caveolae- and clathrin-mediated endocytosis [73], was co-localized with some of the Cav1-eGFP (Figure S4, right panel).

The role of caveolin-mediated endocytosis was further tested by inhibiting Cav1 expression with siRNA. Down-regulation of Cav1 expression was confirmed by immunofluorescent staining and western blotting in Vero cells (Figure 2B, lower right panel). Cav1 down-regulation did not prevent DiI-Ebola VLP internalization (Figure 2B and 2C), upper right panel and Figure 2D), further suggesting that caveolin-mediated endocytosis does not play a critical role in EBOV internalization. Our finding that DiI-labeled EbolaΔVP30 virions enter Cav1-deficient human hepatoblastoma Huh7 cells [74] (Figure 2E) further supports this concept.

Clathrin-, caveolae- and phagocytosis-mediated endocytosis all depend on dynamin 2, a large GTPase that plays an essential role in vesicle scission during clathrin- and caveolae-dependent endocytosis and phagocytosis [75]. Treatment with a dynamine-specific inhibitor, dynasore [76], reduced the internalization of Alexa Fluor-labeled Tf (green; Figure 2F, right panel); however, dynasore did not affect the internalization of DiI-labeled virions (Figure 2F, right panel and Figure 2G). These data indicate that EBOV internalization does not involve clathrin-, caveolin-, or phagocytosis-mediated endocytosis.

### Ebola virions co-localize with sorting nexin (SNX) 5, a component of macropinosomes

Our data argue against a role for clathrin-, caveolae-, or phagocytosis-mediated endocytosis in the internalization of EBOV. We therefore considered macropinocytosis as a potential mode of EBOV entry. Induction of macropinocytosis leads the formation of macropinocytosis-specific endosomes (macropinosomes), which are large enough (0.5–10 µm of diameter) [32] to accommodate Ebola virions.

**Figure 3:**
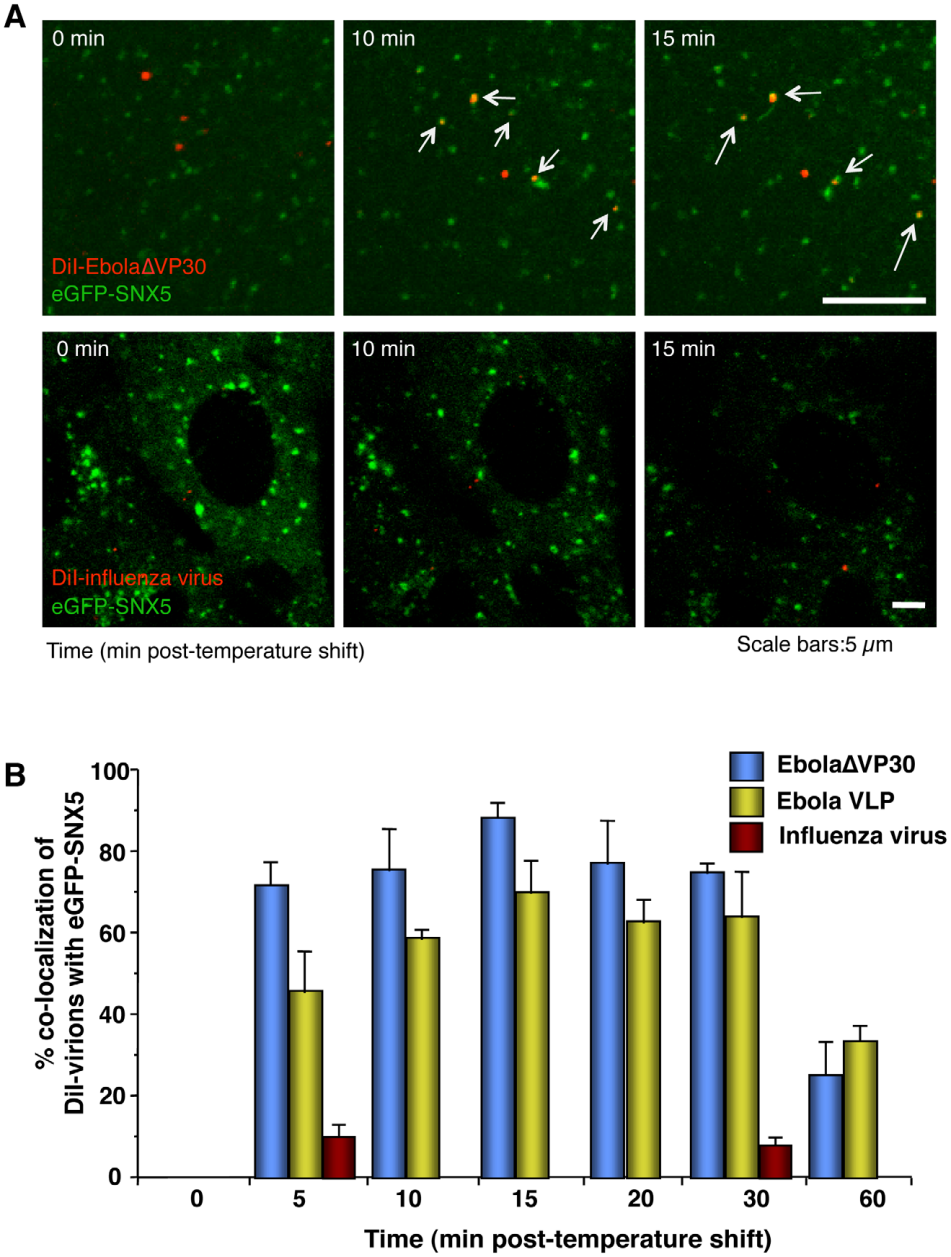
Internalized DiI-EBOV particles co-localize with the macropinosome marker sorting nexin (SNX) 5. (A) Time-lapse analysis of the co-localization of DiI-labeled viral particles with eGFP-SNX5. DiI-EbolaΔVP30 virions (upper panels) or DiI-influenza virus (lower panels) were adsorbed to eGFP-SXN5-expressing Vero cells for 30 min on ice. The cells were then incubated at 37°C and time-lapse images were acquired at 20-second intervals over a period of 20 min by using confocal laser scanning microscope. Still frames at the indicated times (min) after the temperature shift to 37°C are shown. Virions co-localizing with SNX5 are indicated by arrows. Scale bars, 5 µm. (B) Co-localization efficiency of EBOV particles with SNX5. Shown are the co-localization efficiencies of DiI-EbolaΔVP30 (blue bars), DiI-Ebola VLPs (yellow bars), and DiI-influenza virus (red bars) with eGFP-SXN5 at the indicated time points after the temperature shift to 37°C. The number of DiI-labeled virions co-localized with eGFP-SNX5-positive vesicles was measured in 10 individual cells and the percentage of co-localization in the total DiI-virions is shown for each time point. Each experiment was performed in triplicate and the results are presented as the mean ± SD.

Sorting nexin (SNX) 5 comprises a large family of peripheral membrane proteins that associate with newly formed macropinosomes and are involved in their maturation [77], [78]. To assess the role of macropinocytosis in EBOV internalization, we first generated Vero cells expressing an eGFP-SNX5 fusion protein and confirmed that a specific ligand of macropinocytosis, dextran Mw 10,000 (Dex Mw 10K) co-localized with expressed eGFP-SNX5 (Figure S6A) but not with CLCa-eGFP or Cav1-eGFP in Vero cells (Figure S6B). We then asked whether DiI-labeled EbolaΔVP30 and Ebola VLPs co-localize with eGFP-SNX5-positive vesicles. Approximately 70% of DiI-labeled EbolaΔVP30 (blue bars in Figure 3B) and 45% of DiI-labeled Ebola VLPs (yellow bars in Figure 3B) associated with eGFP-SNX5-positive vesicles within 10 min of the temperature shift to 37°C (Figure 3A, upper panels, Figure 3B, and Video S5). Co-localization of viral particles with eGFP-SNX5-positive vesicles continued for 30 min after the temperature shift and then decreased (Figure 3B). On the other hand, DiI-labeled influenza viruses, which are mainly internalized by clathrin-mediated endocytosis [5], did not appreciably co-localize with eGFP-SNX5-positive vesicles (Figure 3A, red bars in lower panels, Figure 3B, and Video S6). We further confirmed co-localization of Ebola VLPs with endogenous SNX5 (Figure S7A). These observations suggest an association of internalized Ebola virions with macropinosomes.

### Internalized DiI-labeled Ebola virions traffic to endosomal compartments

Once internalized, macropinosomes mature into endocytic vesicles [77], [79]. However, the endocytic pathway is also part of the clathrin- and caveolin-mediated entry processes. Several groups have shown that authentic EBOV and EBOV GP-pseudotyped virions enter cells in a low pH- and cathepsin B/L-dependent manner, consistent with endosomal entry [50], [51], [52], [53], [54], [55], [56]. Here, we sought to confirm endosomal localization of EbolaΔVP30 and Ebola VLPs, both of which more closely resemble authentic EBOV than do pseudotyped viruses.

**Figure 4:**
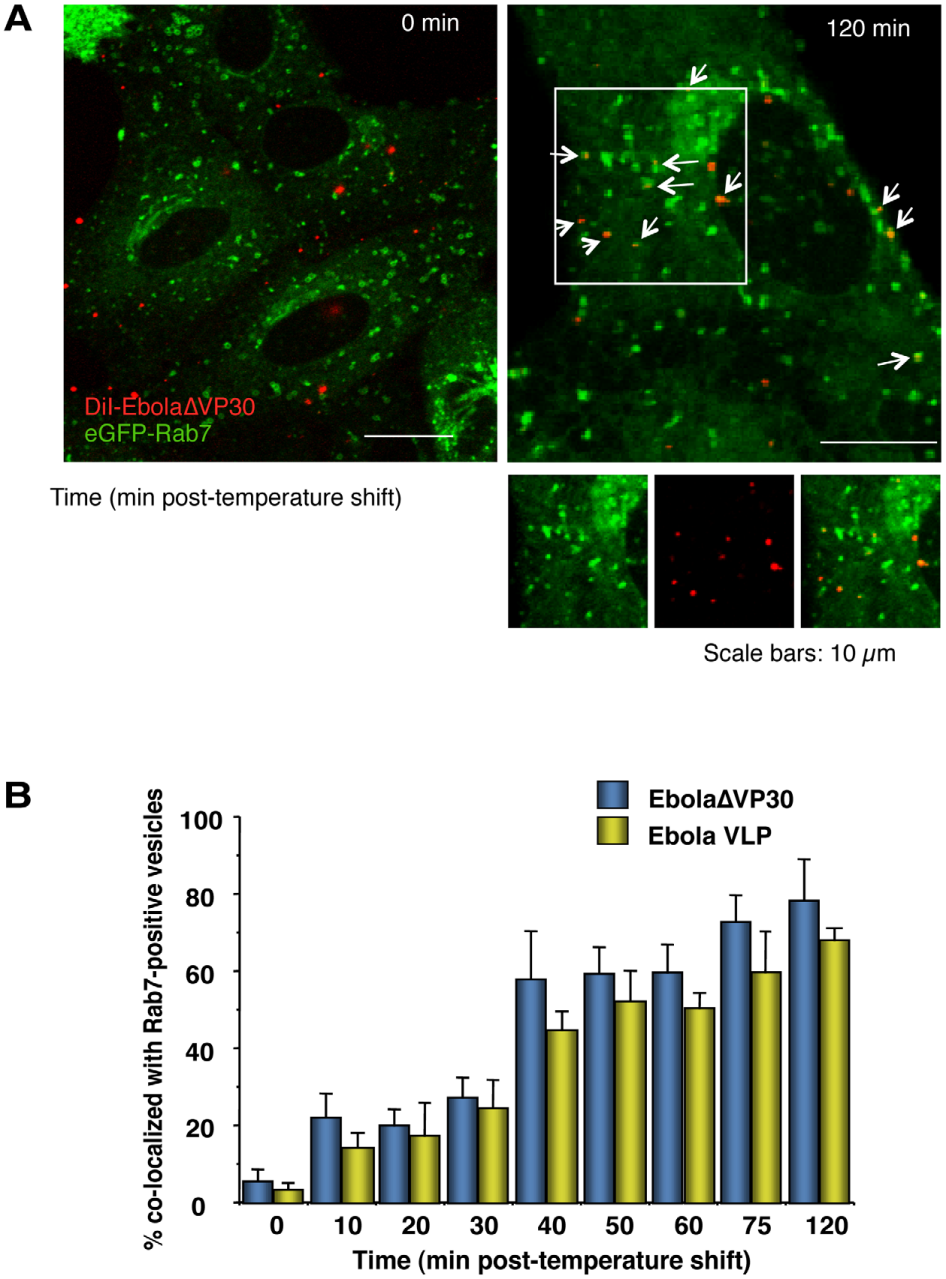
Internalized DiI-labeled EBOV particles are transported to endosomes. (A) Internalized DiI-labeled Ebola virions are transported to Rab7-positive vesicles. DiI-Ebola virions were adsorbed to eGFP-Rab7-expressing Vero cells for 30 min on ice. The cells were then incubated at 37°C and images were acquired at the indicated time points. Shown are representative images at 0 (left panel) and 120 min (right panel) after the temperature shift. DiI-labeled virions that co-localize with Rab7-positive vesicles are indicated by arrows. Insets show enlargements of the boxed areas. Scale bars, 10 µm. (B) Co-localization efficiency of EBOV particles with Rab7-positive vesicles. The co-localization efficiencies of DiI-EbolaΔVP30 virions (blue bars) and -Ebola VLPs (yellow bars) with Rab7-positive vesicles were analyzed at the indicated time points. The number of DiI-labeled virions co-localized with eGFP-Rab7-positive vesicles was measured in 10 individual cells and the percentage of co-localization in the total DiI-virions is shown for each time point. Each experiment was performed in triplicate and the results are presented as the mean ± SD.

The small GTPase Rab7 specifically associates with late endosomes [80], [81] and serves as a marker for this compartment. We, therefore, analyzed the co-localization of internalized DiI-labeled virions with eGFP-Rab7-positive vesicles after the temperature shift. About 20% of DiI-labeled EbolaΔVP30 virions (blue bars in Figure 4B) and Ebola VLPs (yellow bars in Figure 4B) co-localized with eGFP-Rab7 within 10–20 min of the temperature shift; within 2 h of the temperature shift, 70%–80% of EbolaΔVP30 particles and Ebola VLPs co-localized with eGFP-Rab7 (Figure 4A and 4B). Internalized Dex Mw 10K, a specific ligand of macropinocytosis, was also observed in Rab7-positive vesicles (Figure S8). We further confirmed co-localization of Ebola VLPs with endogenous Rab7 (Figure S7B). At 3–4 h after the temperature shift, the DiI-signals were enlarged and overlapped with eGFP-Rab7 (Figure S9, left panel), suggesting fusion of the DiI-labeled viral envelopes with endosomal membranes. Following treatment with NH_4_Cl, which inhibits the acidification of endosomes, the DiI-signals localized with eGFP-Rab7 but remained small (Figure S9, right panel), indicating that NH_4_Cl inhibited membrane fusion. Similarly, VLPs possessing a fusion-deficient GP mutant (F535R) [82] trafficked to eGFP-Rab7-positive vesicles but the signals remained small (Figure S10). Collectively, these findings indicate that internalized EBOV particles are transported to late endosomes, where low pH-dependent membrane fusion occurs.

### Inhibitors of macropinocytosis interfere with EBOV internalization

**Figure 5:**
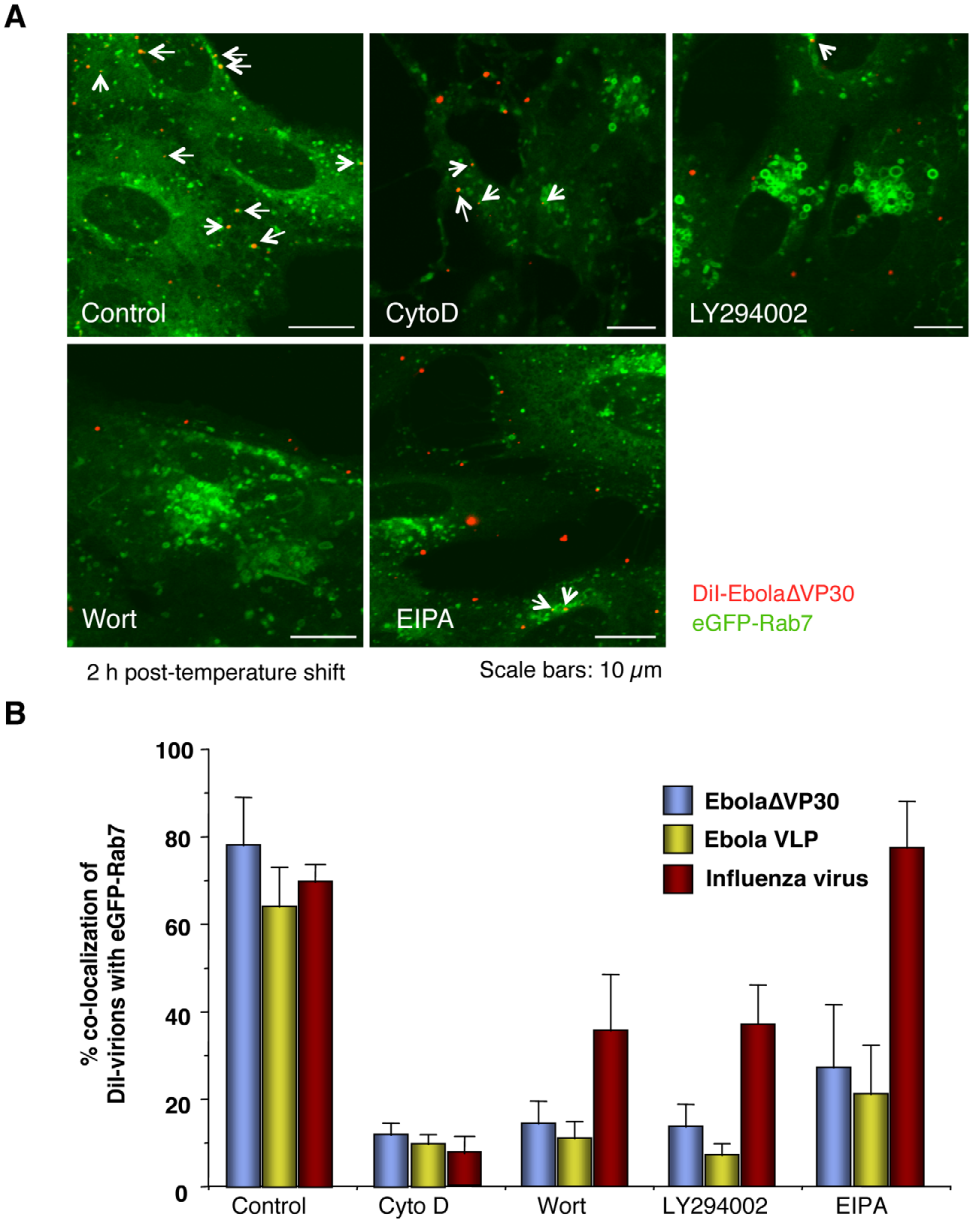
Effect of macropinocytosis inhibitors on the co-localization of DiI-labeled viral particles with Rab7-positive vesicles. Vero cells expressing eGFP-Rab7 were pretreated with cytochalasin D (CytoD), wortmannin (Wort), LY294002, or EIPA for 30 min at 37°C as described in the Materials and Methods. DiI-EbolaΔVP30 virions, DiI-Ebola VLPs and DiI-influenza virus were adsorbed to the cells for 30 min on ice. The cells were then incubated at 37°C in the presence of inhibitors for 2 h. As a control, DMSO-treated cells were incubated with labeled EBOV particles. Representative images of the co-localization of DiI-EbolaΔVP30 virions with eGFP-Rab7 acquired 2 h after the temperature shift are shown (A). DiI-labeled EbolaΔVP30 virions that co-localize with eGFP-Rab7-positive vesicles are indicated by arrows. Scale bars, 10 µm. (B) shows a graphic representation of the data. The number of DiI-labeled EbolaΔVP30 virions (blue bars), Ebola VLPs (yellow bars) and influenza virions (red bars) co-localized with eGFP-Rab7-positive vesicles was measured in 10 individual cells and the percentage of co-localization in the total DiI-virions is shown for each time point. Each experiment was carried out in triplicate and the results are presented as the mean ± SD.

To further test whether Ebola virions are internalized *via* macropinocytosis, we assessed several inhibitors for their effects on EBOV uptake. DiI-labeled influenza virus particles which are internalized *via* clathrin-mediated endocytosis, served as a control. An actin depolymerizing agent, cytochalasin D (CytoD) was used because macropinocytosis depends on actin bundle formation; however, an intact actin skeleton is also critical for other endocytic pathways [83]. Since macropinocytosis relies on PI3K activation [23], [31], we also tested two inhibitors of this kinase, wortmannin (Wort) and LY-294002 [84]. Finally, we used EIPA [5-(N-ethyl-N-isopropyl) amiloride], an inhibitor of the Na^+^/H^+^ exchanger that specifically inhibits macropinocytosis [26], [34], [35], [85], [86]. These inhibitors all inhibited the uptake of Dex Mw 10K (Figure S11A and B). Treatment of cells with the inhibitors appreciably blocked co-localization of EbolaΔVP30 (blue bars in Figure 5B) and VLPs (yellow bars in Figure 5B) with late endosomes, as visualized by eGFP-Rab7 expression (Figure 5A and 5B). CytoD treatment also affected co-localization of DiI-labeled influenza virus with late endosomes (red bars in Figure 5B and S12); this observation was expected because actin is also critical for the internalization of influenza viruses [69], [72], [87]. Entry of influenza virus was also moderately affected by the PI3K inhibitors (Figure 5B and S12), a result consistent with a previous report of PI3K-dependent influenza virus cell entry [88]. However, the internalization of influenza virus was not inhibited by EIPA (Figures 5B and S11), whereas the uptake of DiI-EbolaΔVP30 and Ebola VLPs was appreciably reduced in the presence of this compound (Figure 5A and 5B). These findings suggest that EBOV is internalized *via* macropinocytosis.

### Macropinocytosis-associated events occur during Ebola virion internalization

**Figure 6:**
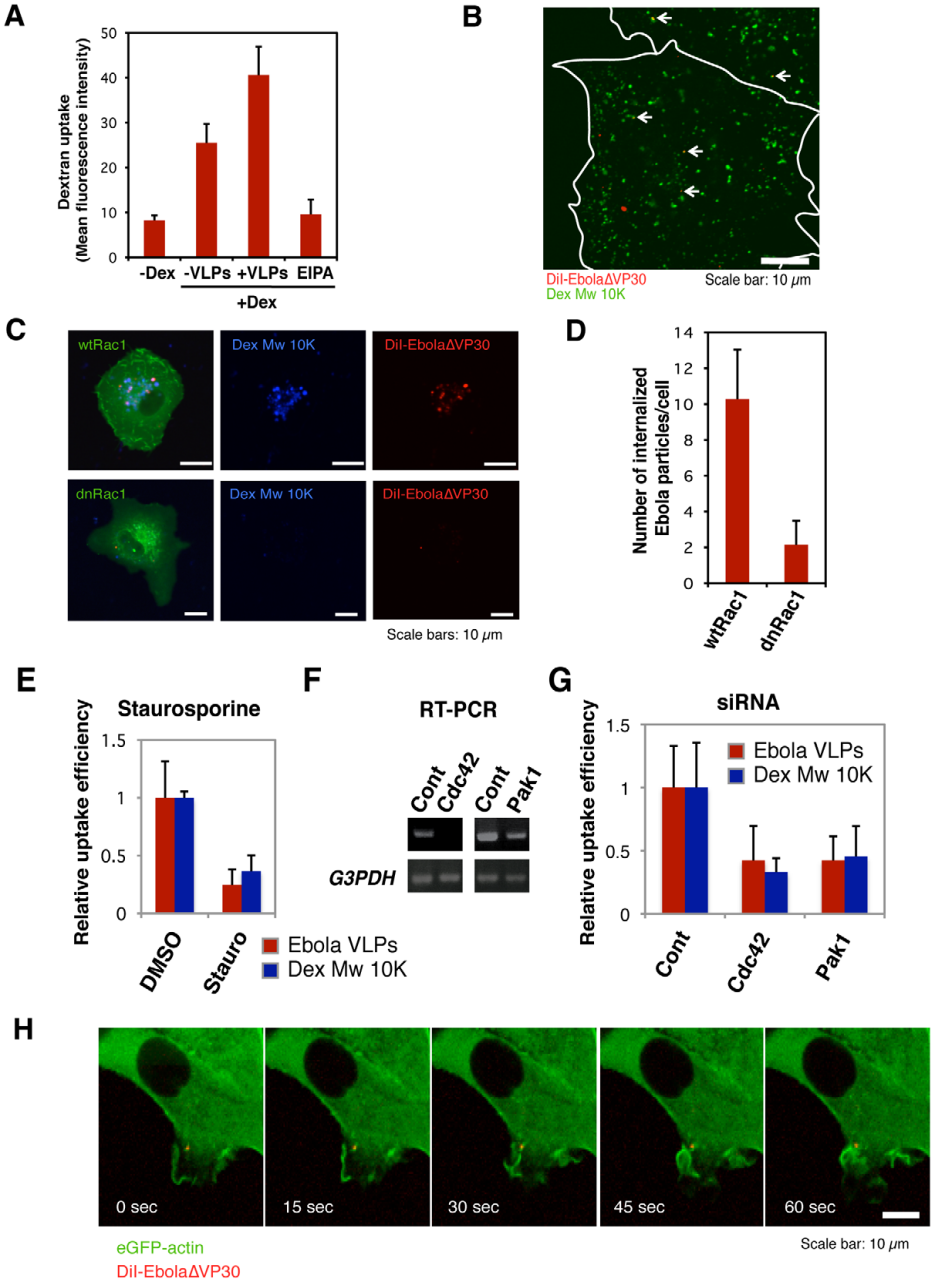
Macropinocytosis-associated events occur during Ebola virion internalization. (A) The effect of the internalization of DiI-labeled Ebola VLPs on dextran uptake. Vero cells were incubated with 0.5 mg/ml Alexa Fluor 647-Dex Mw 10K in the absence or presence of Ebola VLPs for 60 min at 37°C. The uptake of Alexa Fluor 647-Dex Mw 10K was analyzed by using flow cytometry. The effect of EIPA pretreatment was assessed in parallel. Each experiment was performed in triplicate and the results are presented as the mean ± SD. (B) Co-localization of internalized DiI-labeled Ebola VLPs and Dex Mw 10K. DiI-Ebola VLPs were adsorbed to Vero cells for 30 min on ice. The cells were cultured in the presence of 0.5 mg/ml Alexa Fluor 647-Dex Mw 10K for 10 min at 37°C. Co-localization of DiI-virions (red) and Alexa Fluor-Dex Mw 10K (green) was analyzed by using confocal laser scanning microscope. Co-localized virions are shown by arrows. Outlines of individual cells were drawn. Scale bar, 10 µm. (C) Effect of a dominant-negative form of Rac1 on the internalization of DiI-labeled Ebola virions. The eGFP-fused, wild-type Rac1 (wtRac1, upper panels) or the dominant-negative form of Rac1 (dnRac1, lower panels) was expressed in Vero cells. DiI-labeled Ebola VLPs were adsorbed to the cells for 30 min on ice. After incubation for 2 h at 37°C, surface-bound virions were removed by trypsin and the internalization of DiI-virions was analyzed by using confocal laser scanning microscope. Expression of dnRac1 interfered with the internalization of Alexa Fluor 647-Dex Mw 10K (blue; lower middle panel), attesting to its functionality. Scale bars, 10 µm. (D) Quantitative analysis of the internalization of DiI-labeled Ebola virions in wtRac1 or dnRac1-expressed Vero cells. The internalized DiI-virions were measured in 10 individual wtRac1 or dnRac1-expressed cells. Each experiment was performed in triplicate and the results are presented as the mean ± SD. (E) Effect of PKC inhibitors on the internalization of DiI-labeled Ebola virions. Vero cells were treated with DMSO or staurosporine (Stauro) for 30 min at 37°C. Labeled Ebola VLPs were adsorbed to the cells for 30 min on ice and incubated for 2 h at 37°C in the absence or presence of inhibitor. Surface-bound virions were removed by trypsin and the internalization of DiI-virions was analyzed by using confocal laser scanning microscope. The internalized DiI-virions were analyzed in 10 individual DMSO- or staurosporine-treated cells (red bars). The efficiency of Alexa Fluor-Dex Mw 10K uptake in inhibitor-treated cells was measured by using flow cytometry (blue bars). Each experiment was performed in triplicate and relative uptake efficiencies are presented as the mean ± SD (red bars). Staurosporine treatment interfered with the internalization of Alexa Fluor 633-Tf (blue bars), attesting to its functionality. (F) The down-regulation of Cdc42 and Pak1 by siRNA. The efficiencies of Cdc42 and Pak1 knock-down were assessed by use of RT-PCR. Total cellular RNA was isolated from siRNA-transfected Vero cells 48 h post-transfection by using the TRI reagent (Sigma-Aldrich) according to the manufacturer's instructions. cDNA synthesis was performed with Molony murine leukemia virus RTase using a random hexamer (Invitrogen) according to the manufacturer's protocol. PCR was carried out for 25–30 cycles consisting of a DNA denaturing step for 30 s at 94°C, annealing for 30 s at 55°C, and extension for 1 min at 72°C by use of Taq DNA polymerase (Promega). Glyceraldehyde-3-phosphate dehydrogenase (G3PDH) was used as an endogenous control. The oligonucleotides used for amplification of individual genes are shown in Table S1. (G) Effect of down-regulation of Cdc42 and Pak1 on the internalization of DiI-labeled Ebola virions. Vero cells were transfected with control (Cont) non-targeting siRNA or siRNA to down-regulate Cdc42 and Pak1 expression. Labeled Ebola VLPs were adsorbed to the siRNA-transfected cells for 30 min on ice, 48 h post-transfection. After incubation for 2 h at 37°C, surface-bound virions were removed by trypsin for 5 min at 37°C and the internalization of Ebola VLPs was analyzed by using confocal laser scanning microscope, and the number of DiI-virions in 10 individual siRNA-transfected cells was measured. The efficiency of Alexa Fluor-Dex Mw 10K uptake in siRNA-transfected cells was measured by use of flow cytometry (blue bars). Each experiment was performed in triplicate and the relative uptake efficiencies are presented as the mean ± SD. (H) The internalization of Ebola virions is associated with plasma membrane ruffling. DiI-EbolaΔVP30 virions were adsorbed to eGFP-actin-expressing Vero cells for 30 min on ice. The cells were then incubated at 37°C and time-lapse images were acquired at 15-second intervals over a period of 10 min by using confocal laser scanning microscope. Still frames at the indicated times (sec) after the temperature shift to 37°C are shown. Scale bar, 10 µm.

Constitutive macropinocytosis occurs in specific cell types such as dendritic cells and macrophages [18], [19], [20]; however, in epithelial cells, it is initiated in response to growth factor stimulation [21], [22] or expression of ruffling kinases [23], [24], [25]. To assess whether Ebola virions activate macropinocytosis to allow EBOV be internalized into the cells, we asked whether the virions accelerated the uptake of a macropinocytosis marker, Dex Mw 10K. In the presence of Ebola virions, the uptake of Dex Mw 10K was accelerated (Figure 6A and S13), and this event was inhibited by EIPA. Co-localization of DiI-EbolaΔVP30 and Alexa Fluor-Dex Mw 10K was also observed (Figure 6B).

The Rho GTPases (Rac1 and Cdc42), protein kinase C (PKC), and Pak1 are involved in the regulation of macropinocytosis[23], [24], [27], [29], [30]. Therefore, we examined the role of Rac1 by use of dominant-negative Rac1 (dnRac1) [89]. Expression of eGFP-fused dnRac1 inhibited the internalization of Ebola virions (red) into cells by 80% (Figure 6C, lower right panel; Figure 6D) compared with that of eGFP-fused wild-type Rac1 (wtRac1) (Figure 6C upper right panel; Figure 6D). dnRac1 expression also interfered with the uptake of Dex Mw 10K (blue) (Figure 6C, lower middle panel), indicating that expression of dnRac1 inhibited macropinocytosis. The role of PKC in the internalization of Ebola virions was tested by use of the specific PKC inhibitor staurosporine [90]. Staurosporine reduced the internalization of DiI-virions (red bars in Figure 6E and left panels in Figure S14A) and Dex Mw 10K (blue bars in Figure 6E and right panels in Figure S14A) by 80% and 70%, respectively. The effect of down-regulation of Cdc42, and Pak1 by siRNAs on Ebola VLP uptake was also tested. Down-regulation at the mRNA level was assessed by RT-PCR (Figure 6F). Knockdown of Cdc42 and Pak1 appreciably interfered with DiI-Ebola VLP internalization (red bars in Figure 6G and left panels in Figure S14B) and also reduced the uptake of Dex Mw 10K (blue bars in Figure 6G and right panels in Figure S14B). Since plasma membrane ruffling precedes macropinocytosis [14], [15], [16], we monitored ruffling formation in the internalization of DiI-virions by use of Vero cells expressing eGFP-actin [91]. Time-lapse analysis revealed that prominent plasma membrane ruffling was associated with DiI-labeled virions after the temperature-shift (Figure 6H and Video S7). Appreciable actin rearrangement was not observed in the absence of EBOV virions (Figure S15 and Video S8). Together, these results demonstrate that Ebola virions stimulate macropinocytosis along with the activation of the cellular factors involved in actin polymerization that allow the virions to be internalized.

### Internalization of EBOV particles is GP-dependent

**Figure 7:**
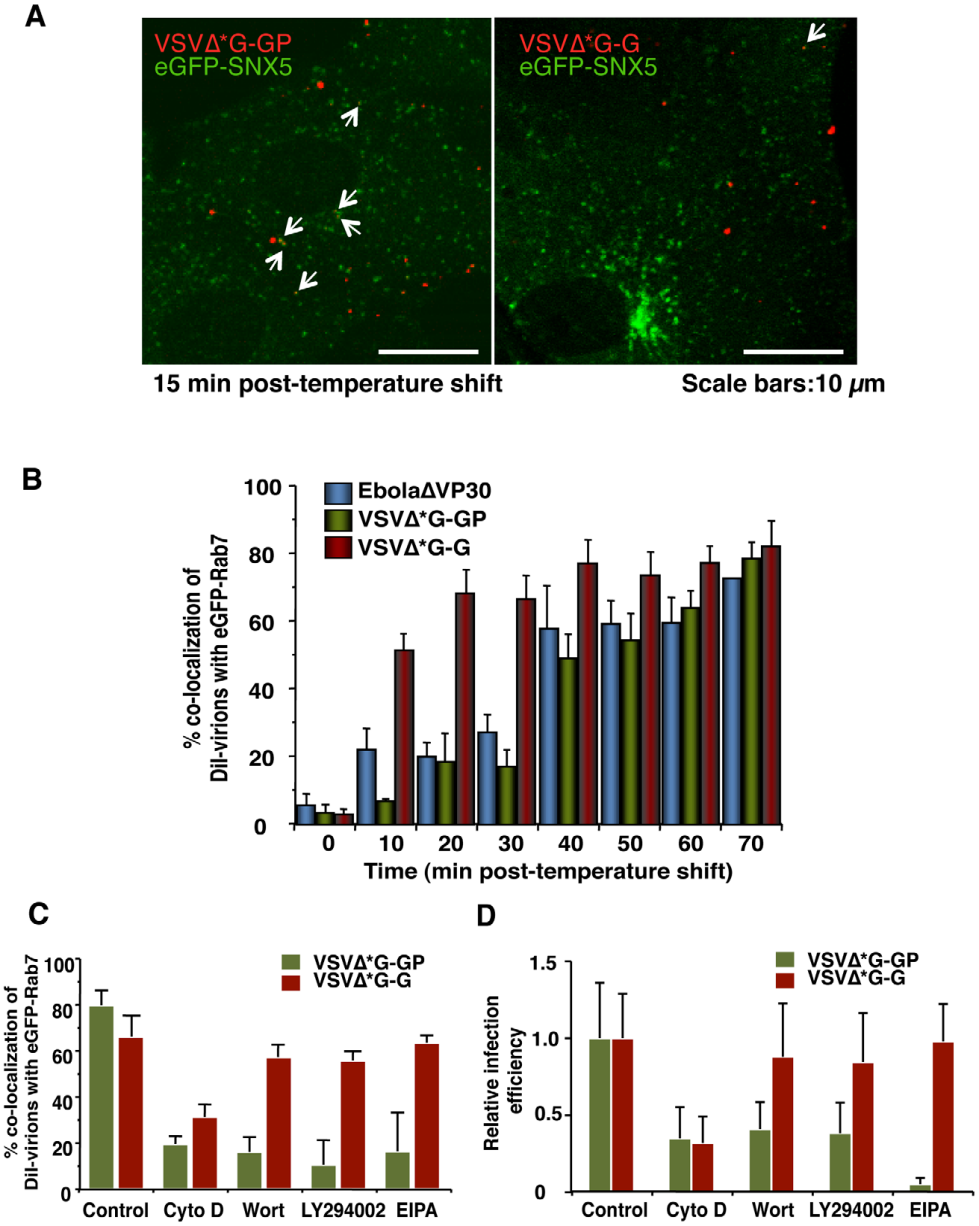
Macropinocytotic internalization of Ebola virions is GP-dependent. (A) Co-localization of SNX5 with VSV pseudotyped with EBOV GP. Labeled VSV particles pseudotyped with EBOV GP (DiI-VSVΔ*G-GP) or VSV G (DiI-VSVΔ*G-G) were adsorbed to eGFP-SNX5-expressing Vero cells for 30 min on ice. The cells were then incubated at 37°C and time-lapse images were acquired at 20-second intervals over a period of 30 min by using confocal laser scanning microscope. Still frames of DiI-VSVΔ*G-GP (left panel) and DiI-VSVΔ*G-G (right panel) at 10 min after the temperature shift are shown. DiI-pseudovirions that co-localize with eGFP-SNX5 are indicated by arrows. Scale bars, 10 µm. (B) Graphic representation of the co-localization of EBOV GP-pseudotyped VSV virions with Rab7-positive vesicles. Co-localization of DiI-VSVΔ*G-GP (green bars) with Rab7-positive vesicles was analyzed at the indicated time points as indicated in the Materials and Methods. Experiments were performed in triplicate and the results are presented as the mean ± standard deviation. Results obtained for DiI-EbolaΔVP30 (blue bars) and DiI-VSVΔ*G-G (red bars) are shown for comparison. (C) Effect of macropinocytosis inhibitors on the co-localization of DiI-labeled VSV pseudovirions with eGFP-Rab7-positive vesicles. Vero cells expressing eGFP-Rab7 were pretreated with CytoD, Wort, LY294002 or EIPA for 30 min at 37°C; control cells were treated with DMSO. DiI-labeled VSVΔ*G-GP (green bars) or VSVΔ*G-G (red bars) were adsorbed to cells for 30 min on ice. The cells were then incubated at 37°C in the presence of inhibitors for 2 h. Co-localization of DiI-pseudovirions with eGFP-Rab7-positive vesicles was analyzed as described in the Materials and Methods. Experiments were carried out in triplicate and the results are presented as the mean ± standard deviation. (D) Effect of macropinocytosis inhibitors on the infectivity of VSV pseudovirions. Vero cells were treated with individual inhibitors for 30 min at 37°C and infected with VSVΔG*-GP (green bars) or VSVΔG*-G (red bars) in the presence of the inhibitor. 1 h post-infection, surface-bound virions were removed by trypsin and the cells were cultured for 24 h in the absence of inhibitors. The infection efficiency of each pseudovirus was determined by measuring the number of GFP-positive cells using with conventional fluorescent microscope. Each experiment was performed in triplicate and the relative infection efficiencies are presented as the mean ± SD.

Our data indicate that the EBOV particle internalization occurs *via* macropinocytosis, whereas previous studies suggest that clathrin- or caveolin-dependent endocytosis mediate the internalization of wild-type EBOV and EBOV GP-pseudotyped VSV or retroviruses [50], [57], [58], [59]. To determine if these conflicting findings result from differences in assay systems (i.e., use of pseudotype viruses) and/or experimental conditions used, we tested whether a VSV pseudotyped with EBOV GP (VSVΔ*G-GP) was internalized by macropinocytosis. Although VSV is known to be internalized *via* the clathrin-dependent pathway [6], DiI-labeled VSVΔ*G-GP virions did not co-localize with CLCa-eGFP or Cav1-eGFP (Figure S16), whereas DiI-labeled VSVΔ*G-G virions co-localized with CLCa-eGFP (Figure S17). By contrast, DiI-labeled VSVΔ*G-GP virions co-localized with eGFP-SNX5 (Figure 7A, left panel), indicative of macropinocytosis. No significant co-localization with eGFP-SNX5 was observed for a DiI-labeled control virion possessing the VSV G glycoprotein (DiI-VSVΔ*G-G; Figure 7A, right panel). EbolaΔVP30 particles possessing authentic morphologies (blue bars in Figure 7B) and VSV pseudotyped with EBOV GP (green bars in Figure 7B) co-localized with eGFP-Rab7-positive vesicles with similar kinetics, indicating that the smaller size of the VSV virions relative to that of the Ebola virions did not affect the kinetics of internalization. The kinetics of DiI-VSVΔ*G-G trafficking to late endosomes/lysosomes (red bars in Figure 7B) was consistent with a previous study of authentic VSV [92]. EIPA, which specifically interferes with macropinocytosis, blocked the co-localization of eGFP-Rab7 with DiI-labeled VSVΔ*G-GP (green bars in Figure 7C), but not with DiI-VSVΔ*G-G (red bars in Figure 7C). The PI3K inhibitors significantly reduced the co-localization of eGFP-Rab7 with DiI-labeled VSVΔ*G-GP (green bars in Figure 7C) but not with VSVΔ*G-G (red bars in Figure 7C), which is consistent with previous findings [93].

The effect of these inhibitors was further assessed in a viral infection system by use of a VSV pseudovirion encoding eGFP. Vero cells were pre-treated with one of the inhibitors and then infected with VSVΔG*-GP (green bars in Figure 7D) or VSVΔG*-G (red bars in Figure 7D) in the presence of the inhibitors. The infection efficiency of each pseudovirus was determined by measuring the number of GFP-positive cells. EIPA blocked the infection of VSVΔ*G-GP (green bars in Figure 7D), but not VSVΔ*G-G (red bars in Figure 7D). The PI3K inhibitors reduced the infection of VSVΔ*G-GP (green bars in Figure 7D) but not VSVΔ*G-G (red bars in Figure 7D), which is consistent with the results of the co-localization of DiI-VSV pseudovirions and SNX5 (Figure 7C). These findings demonstrated that in this viral infection system, VSV pseudotyped with EBOV GP is internalized by macropinocytosis, as are EbolaΔVP30 and Ebola VLPs. Therefore, regardless of the size of the virions, our data indicate that EBOV GP induces receptor-dependent macropinocytosis, unlike those in a previous report which showed that macropinocytosis is receptor-independent [32]. Our finding is consistent with a recent report describing receptor-dependent macropinocytosis in adenovirus type 3 [33].

## Discussion

Viruses accomplish cell entry by hijacking the cellular endocytic machinery. In this study, with EBOV particles that resemble authentic EBOV, the data lead us to conclude that EBOV is internalized into host cells *via* macropinocytosis in a viral GP-dependent manner.

**Figure 8:**
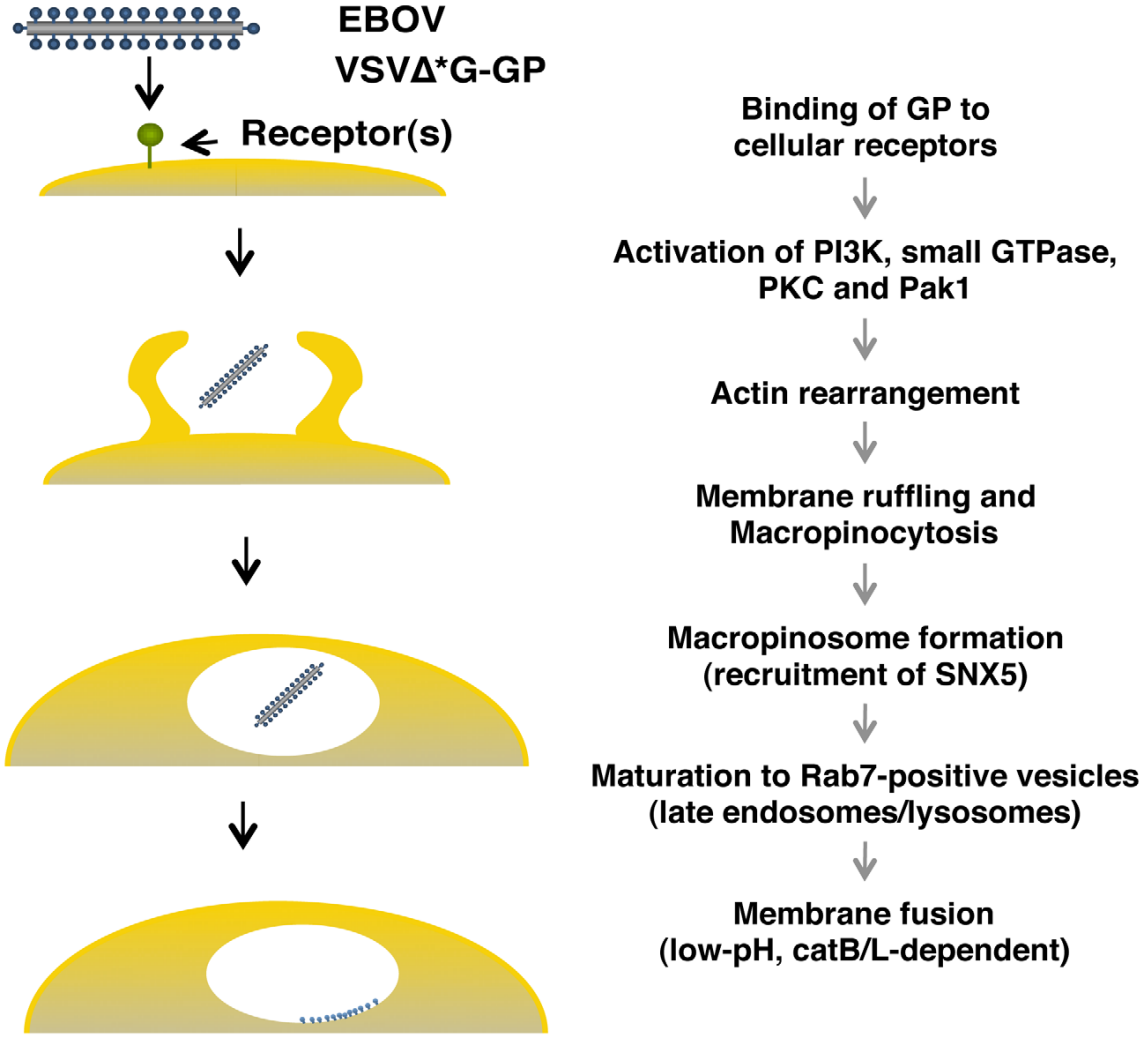
Model of GP-dependent EBOV cell entry. For EBOV cell entry, the binding of GP to cellular receptor(s) may activate cellular actin modulators (PI3K, small GTPases, PKC and Pak1), which trigger the actin-dependent membrane ruffling that leads to macropinocytosis. The virions are then internalized *via* macropinocytosis. Macropinosomes containing the virions are eventually fused to Rab7-positive late endosomes/lysosome (late maturation), resulting in the fusion of the viral envelope with the endosomal membrane in a low pH- and cathepsin-dependent manner.

Our conclusion that EBOV is internalized *via* macropinocytosis is based on the following observations: (i) the internalized viral particles co-localize with a marker of macropinosomes, SNX5 (Figure 3); (ii) the internalization of viral particles was blocked by inhibitors of actin polymerization and PI3K, which are known players in macropinocytosis and also by a specific inhibitor of macropinocytosis, EIPA (Figure 5); (iii) the internalization of Ebola virions accelerated the uptake of a specific cargo for macropinosomes Dex Mw 10K (Figure 6A) and the internalized virions co-localized with Dex Mw 10K (Figure 6B); (iv) the internalization of viral particles was blocked by a dominant-negative Rac1 (Figure 6C and 6D), a PKC inhibitor (Figure 6E) and the down-regulation of Cdc42 and Pak1 (Figure 6F and 6G); and (v) the internalization of viral particles was associated with membrane ruffling (Figure 6H). These findings suggest a model in which the binding of EBOV glycoprotein to cellular receptor(s) activates multiple macropinocytosis inducers (PI3K, Rac1, PKC, Cdc42, and Pak1), triggering plasma membrane ruffling and macropinocytosis (Figure 8). Internalized Ebola virions then traffic to Rab7-positive late endosomes/lysosomes (Figure 4), where membrane fusion occurs (Figure 8).

Two findings, the inability to enter cells of Ebola VLPs lacking GP (Figure S2) and the macropinocytic uptake of VSV particles pseudotyped with EBOV GP (Figure 7), support a role for GP in the macropinocytic internalization of EBOV particles. Macropinocytosis was thought to be receptor-independent [32] until a recent study showed that Ad3 entry *via* macropinocytosis requires receptors (CD46 and integrins) [33]. This finding, together with our observations, supports the concept of receptor-mediated macropinocytic pathways. The exact mechanism of GP-mediated macropinocytosis remains to be elucidated; however, mannose-binding lectin, a potential EBOV co-receptor [43], is known to accelerate macropinocytosis and phagocytosis for the uptake of apoptotic cells and bacteria into macrophages [94], [95]. In addition, integrins, which are also potential EBOV co-receptors [47], play an important role in Ad3 entry *via* macropinocytosis [33]. Thus, macropinocytosis is likely initiated through GP interaction with EBOV co-receptors on the cell surface (Figure 8).

Recently, one study demonstrated that the entry of Ebola VLPs and pseudovirions depends on the PI3K-Akt signaling pathway and Rac1 [93]. PI3K and its effectors are responsible for ruffling and macropinocytosis [23], [31]. Rac1 is also critical for the induction of actin filament accumulation at the plasma membrane, which leads to membrane ruffling and macropinocytosis [27]. Moreover, membrane-bound Rac1 localizes to macropinosomes [26], [27], [28]. Other study demonstrated that overexpression of RhoC GTPase facilitated wild-type EBOV entry and VSV pseudotyped with EBOV GP [96]. Although a role of RhoC in viral entry has not been specifically characterized, the overexpression of RhoC resulted in increased dextran uptake and in formation of increased actin organization [96], suggesting that RhoC plays a role in EBOV entry mediated *via* macropinocytosis.

Taken together with our findings, these observations support the model of EBOV entry through macropinocytosis.

Clathrin-mediated endocytosis was thought to contribute to EBOV entry based on findings that specific inhibitors of clathrin-mediated endocytosis blocked the expression of viral antigens in EBOV-infected cells [59]. However, some of these inhibitors caused severe cytotoxicity, which may have induced the down-regulation of viral antigen expression [59]. Recently, by using specific inhibitors of clathrin-mediated endocytosis, a dominant-negative Eps15, which abrogates CCP formation, and siRNA for CHC, a possible role for the clathrin-dependent pathway in the internalization of retrovirus pseudovirions with EBOV GP was suggested [60]. The discrepancy between this study and ours may originate from the difference in pseudotype systems (retrovirus versus VSV or Ebola virions) and specific cell types [60]. Our data demonstrate that down-regulation of cellular CHC, which specifically blocks clathrin-mediated endocytosis, does not interfere with the internalization of Ebola virions which resemble authentic EBOV in their morphology into Vero cells (Figure 1B).

Caveolae- and lipid-raft-mediated endocytosis were also thought to play a role in EBOV entry because FR-α, a potential co-receptor of filovirus entry, localizes to lipid rafts and is internalized through lipid raft-associated caveolae [40]. However, the role of FR-α in EBOV entry remains controversial [51], [97]. The internalization of EBOV GP-pseudotyped virions was sensitive to the depletion of cholesterol, a major component of caveolae and lipid rafts [50], [57], [58]; however, cholesterol is also required for membrane ruffling and macropinocytosis [98]. Moreover, the internalization of Ebola virions into cells transfected with siRNA for Cav1 (Figure 2B and 2C) or that lacked Cav1 (Figure 2D), argues against a role for caveolae-mediated endocytosis in EBOV entry.

One study [59] ruled out macropinocytic uptake of wild-type EBOV based on the use of an amiloride; however, the concentration of the drug used was one tenth of that typically used and may not have allowed the authors to detect an effect of this anti-macropinocytic drug on EBOV internalization.

After internalization, EBOV particles traffic to late endosomes, as suggested by their co-localization with Rab7-positive vesicles (Figure 4). This finding is consistent with previous studies that identified low pH- and cathepsin B/L-requirements for the internalization of EBOV and pseudovirions with EBOV GP into host cells [50], [51], [52], [53], [54], [55], [56].

Currently, no antivirals or vaccines are available for EBOV infections. Since viral entry is an attractive target for therapeutic intervention, it is imperative that we understand the mechanism of EBOV cell entry. Our finding that EBOV is likely internalized through macropinocytosis may stimulate the development of compounds that interfere with the EBOV internalization process.

## Materials and Methods

### Plasmids and reagents

Human CLCa, Cav1, and Rab7 genes were amplified by RT-PCR from total RNA derived from HeLa cells, and subcloned into pEGFP-N1 or pEGFP-C1 plasmids (Clontech, Mountain View, USA). The eGFP-SNX5 and eGFP-actin expression plasmid was a kind gift from Drs Rohan D. Teasdale (University of Queensland, Brisbane, Australia) and David Knecht (University of Connecticut), respectively. The eGFP-fused genes were cloned into a moloney murine leukemia virus-based retrovirus plasmid [99], a kind gift from Dr. Bill Sugden (University of Wisconsin-Madison, Madison, USA). Expression plasmids for eGFP-fused wild-type and dominant-negative Rac1 were purchased from Addgene (Cambridge, USA). DiI, Alexa Fluor 633-labeled Tf and Alexa Fluor 647-labeled Dex Mw 10K were purchased from Invitrogen (Carlsbad, USA). Dynasore, Cytochalasin D, Wortmannin, LY-294002 hydrochloride, EIPA, and Staurosporine were purchased from Sigma-Aldrich (St. Louis, USA). Antibodies for human clathrin heavy chain and Caveolin 1 were purchased from Abcam (Cambridge, UK).

### Cell culture and transfection

African green monkey kidney epithelial Vero cells were grown in minimum essential medium (MEM) supplemented with 10% fetal bovine serum (FBS), L-glutamine, vitamins, nonessential amino acids, and antibiotics. A Vero cell line stably expressing the EBOV VP30 protein [62] was maintained in complete MEM containing 5 µg/ml puromycin (Sigma-Aldrich). Human embryonic kidney 293T cells and human hepatoblastoma cell line Huh7 cells were grown in high-glucose Dulbecco's modified Eagle's medium (DMEM) containing 10% FBS and antibiotics. Cells were maintained at 37°C in 5% CO_2_. Plasmid transfections in Vero cells were carried out with FuGENE HG (Roche, Basel, Switzerland).

### Retroviral infection

Recombinant retroviruses for the expression of CLCa-eGFP, Cav1-eGFP, eGFP-SNX5, -actin and -Rab7, were produced and purified as previously described [99]. For retroviral infections, Vero cells were grown to 20%–30% confluence, at which point the culture medium was replaced with ice-cold MEM supplemented with 10% FBS and 20 mM Hepes (pH 7.4), and the cells were incubated with viral stocks (10^7^–10^8^ infectious units/ml) for 1 h at 4°C at a multiplicity of infection (m.o.i) of 5. After being washed twice with complete medium, the cells were cultured in complete medium for 48 h.

### Purification and fluorescent-labeling of viral particles

For the purification of EbolaΔVP30, Vero cells stably expressing VP30 were infected with EbolaΔVP30 stock [62] at a m.o.i of 0.1 in MEM containing 4% BSA and 2% FBS. EbolaΔVP30-containing culture medium was harvested 5 days post-infection and centrifuged at 3,500 rpm for 15 min to remove cell debris. The virions were precipitated through a 30% sucrose cushion by centrifugation at 11,000 rpm for 1 h at 4°C with an SW28 rotor (Beckman, Fullerton, USA). Precipitated virions were resuspended in TNE buffer [10 mM Tris-HCl (pH 7.6), 100 mM NaCl, 1 mM EDTA], and fractionated by use of a 2.5%–30% Nicodenz (Nycomed Pharma AS, Oslo, Norway) gradient in TNE buffer at 27,000 rpm for 2.5 h at 4°C with an SW40 rotor (Beckman). The purification efficiency was confirmed by Coomassie Brilliant Blue staining and western blot analysis with antibodies to VP40 and NP. The infectious titer was determined by plaque assay, as described previously [62].

For purification of Ebola VLPs, equal amounts of the expression plasmids for EBOV VP40 [100], [101], NP [100], and GP [100], [101] were transfected into 293T cells by using TransIT LT-1 (Mirus, Madison, USA). Forty-eight hours post-transfection, the culture supernatants were harvested and released VLPs were purified, as described above. Incorporation of viral proteins in the purified VLPs was confirmed by western blot analysis with antibodies to VP40, GP and NP, and the morphology of the VLPs was confirmed by negative staining (Figure S3).

Influenza virus A/PR/8/34 was prepared and purified as described previously [102]. VSV pseudotyped with EBOV GP (VSVΔG*-GP) was generated as described previously [61] and purified as described above. Protein concentrations of the individual virion fractions were measured by use of a Bradford protein assay kit (BioRad, Hercules, USA).

Viral particles were fluorescently labeled as described by Sakai *et al.* [67]. Briefly, 1 ml of fractionated virions (100 µg/ml) was incubated with 6 µl of 100 µM stock solution of DiI in the dark for 1 h at room temperature with gentle agitation.

### Imaging of the internalization of DiI-labeled viral particles in live cells

For real-time imaging of the internalization of DiI-labeled viral particles, Vero cells expressing CLCa-eGFP, Cav1-eGFP, eGFP-SNX5, eGFP-actin or eGFP-Rab7 were cultured in 35 mm glass-bottom culture dishes (MatTek corporation, Ashland, USA), washed in 1 ml of phenol red-free MEM (Invitrogen) containing 2% FBS and 4% BSA, and incubated with DiI-labeled virions in 50 µl of the same medium on ice for 30 min. The cells were washed with the ice-cold medium and incubated for various times in a temperature-controlled chamber on the stage of a confocal laser scanning microscope (LSM510 META, Carl Zeiss, Oberkochen, Germany); the chamber was maintained at 37°C with a humidified atmosphere of 5% CO_2_. Images were collected with a 40x oil objective lens (C-Apochromat, NA = 1.2, Carl Zeiss) and acquired by using LSM510 software (Carl Zeiss). For presentation in this manuscript, all images were digitally processed with Adobe Photoshop. For co-localization analysis, the images were acquired randomly, the number of DiI-labeled virions that co-localized with eGFP-SNX5 or eGFP-Rab7-positive vesicles were measured in 10 individual cells (approximately 10–20 dots/cell), and the percentage of co-localization in the total DiI-virions was determined for each time point. Each experiment was performed in triplicate and the results are presented as the mean ± standard deviation.

### siRNA treatment

Target sequences corresponding to the human CHC [103], Cav1 [104],and Cdc42 [105] -coding sequences were selected, respectively (Table S1), and synthesized (Dharmacon, Lafayette, USA or Qiagen, Hilden, Germany). siRNA for Pak1 down-regulation was purchased from Cell Signaling (Trask Lane, USA). Synthesized siRNA was transfected into Vero cells by using TransIT-TKO (Mirus, Madison, USA). For analysis of the efficiencies of internalization of Ebola virions, DiI-virions were adsorbed to the siRNA-transfected cells 48 h post-transfection, as described above, and then incubated for 2 h at 37°C. Uninternalized surface-bound virions were removed by the addition of 0.25% trypsin for 5 min at 37°C and the number of DiI-virions in 10 individual cells was counted. Each experiment was performed in triplicate and the results are presented as the mean ± SD.

The efficiency of CHC and Cav1 down-regulation was assessed by immunofluorescent staining with antibodies specific to CHC and Cav1 (Abcam, Cambridge, UK). The down-regulation of endogenous Cav1 was also examined by western blot analysis by using an antibody specific to Cav1 (Abcam). The efficiencies of Cdc42 and Pak1 [106] were assessed by RT-PCR with oligonucleotides to amplify each gene (Table S1).

### Inhibitor treatment

Vero cells or Vero cells expressing eGFP-Rab7 cultured in 35 mm glass-bottom culture dishes were pretreated with 100 µM dynasore (Sigma-Aldrich), 2 µM cytochalasin D (Sigma-Aldrich), 50 µM LY294002 hydrochloride (Sigma-Aldrich), 50 nM wortmannin (Sigma-Aldrich), 100 µM EIPA (Sigma-Aldrich) or 100 nM staurosporine (Sigma-Aldrich) for 30 min at 37°C. DiI-labeled virions were adsorbed to the cells for 30 min on ice in the presence of these inhibitors in phenol red-free MEM (Invitrogen) containing 2% FBS and 4% BSA. Cells were then washed with the same medium and incubated for 2 h at 37°C in the presence of the inhibitors. As a control, cells were treated with dimethyl sulfoxide (DMSO, Sigma-Aldrich). Efficiencies of internalization of DiI-labeled viral particles into Vero cells or co-localization of DiI-labeled viral particles with eGFP-Rab7 were analyzed by using confocal laser scanning microscope as described above.

### Transferrin and dextran uptake assays

Vero cells treated with dynasore, transiently expressing dominant-negative Rac1, were incubated with DiI-labeled virions on ice for 30 min in MEM containing 2% FBS and 4% BSA. The cells were washed with the same medium and then incubated for 2 h at 37°C. Cells were then incubated with 2 µg/ml Alexa Fluor 633-Tf for 10 min or 0.5 mg/ml Alexa Fluor 647-Dex Mw 10K for 60 min at 37°C. To remove surface-bound labeled virions, Tf, or Dex Mw 10K, the cells were treated with trypsin as described above for 5 min at 37°C. Cells were then washed twice with the same medium and internalized DiI-labeled virions, Tf or Dex Mw 10K were analyzed by use of confocal laser scanning microscope. To assess the effect of staurosporine or the siRNA treatment on fluid phase uptake, after staurosporine pretreatment or 48 h post-transfection of individual siRNAs, Vero cells were incubated with 0.5 mg/ml AlexaFluor 647-Dex Mw 10K, harvested by treating with trypsin, washed twice with ice-cold PBS, and fixed with 4% PBS-buffered paraformaldehyde for 10 min at room temperature. The mean fluorescence intensities in the cells were analyzed by use of flow cytometry (FACSCalibur; Becton Dickinson, Franklin Lakes, USA).

### VSV pseudovirion infectivity analysis

VSV pseudotyped with EBOV GP (VSVΔG*-GP) or VSV G (VSVΔG*-G) expressing GFP was generated as described previously [61]. Vero cells were treated with a series of inhibitors for 30 min at 37°C and infected with each virus at a multiplicity of infection (as titrated with Vero cells) of 0.002 to 0.005 in the presence of the inhibitors. 1 h post-infection, surface-bound virions were removed by trypsin as described above and cultured for another 24 h. Infection efficiencies for VSVΔG*-GP or VSVΔG*-G were determined by measuring the number of GFP-positive cells by conventional fluorescent microscope. Each experiment was performed in triplicate and the results are presented as the mean ± SD.

### Methods for supporting information files

Methods for supporting information files are described in Text S1.

## Supporting Information

Figure S1 The effect of adsorption temperature on Ebola virion internalization. DiI-labeled Ebola VLPs were adsorbed to Vero cells grown in 35 mm glass-bottom culture dishes for 30 min on ice (4°C), room temperature (r.t.), or 37°C in parallel. The cells were then incubated for 2 h at 37°C. Surface-bound virions were removed by trypsin and the internalization of the DiI-virions was measured in 10 individual cells by use of confocal laser scanning microscope. Each experiment was performed in triplicate and the results are presented as the mean ± SD.
(3.40 MB TIF)

Figure S2 Visualization of the internalization of DiI-labeled EBOV particles in live cells. (A) DiI-labeled Ebola-VLPs (red; left panel) or control VLPs lacking GP [(Ebola VLPs (-GP)] (red; right panel) were absorbed to Vero cells for 30 min on ice. The cells were incubated at 37°C and time-lapse images were acquired at 20-second intervals over a period of 20 min by using a confocal laser scanning microscope. Still frames at the indicated times (min) after the temperature shift to 37°C are shown. Individual cells are highlighted. Initial positions of individual viral particles are shown as white dots. Scale bars, 10 µm. (B) DiI-labeled Ebola VLPs (red; left panel) or Ebola VLPs (-GP) (red; right panel) were absorbed to Vero cells for 30 min on ice. The cells were then incubated for 30 min at 37°C. Images were collected by taking 10∼15 optical slices of z-stack in 0.16 µm steps and the cross-sectional views were processed with LSM510 software. Outlines of individual cells were drawn. Scale bars, 10 µm.
(1.04 MB TIF)

Figure S3 Filamentous morphologies of Ebola VLPs. Ebola VLPs released into the supernatants of 293T cells expressing EBOV VP40, NP and GP were purified as described in the Materials and Methods and then negatively stained with 1% uranyl acetate. Filamentous particles of various lengths with surface spikes can be seen. Scale bar, 1 µm.
(3.91 MB TIF)

Figure S4 Transferrin and cholera toxin subunit B are co-localized with CLCa-eGFP and Cav1-eGFP, respectively. (Left panel) Vero cells expressing CLCa-eGFP were incubated with 2 µg/ml Alexa Fluor 594-Transferrin (Tf) (red) for 30 min on ice. The cells were then incubated for 3 min at 37°C and subsequently fixed in PBS-buffered 4% paraformaldehyde. The co-localization of Alexa Fluor-Tf with CLCa-eGFP was analyzed by using confocal laser scanning microscope. The inset shows an enlargement of the boxed area. Scale bar, 1 µm. (Right panel) Vero cells expressing Cav1-eGFP were incubated with 2 µg/ml Alexa Fluor 647-cholera toxin subunit B (CtxB) (purple) for 30 min on ice. The cells were then incubated for 60 min at 37°C and subsequently fixed in PBS-buffered 4% paraformaldehyde. The co-localization of Alexa Fluor-CtxB with Cav1-eGFP was analyzed by use of a confocal laser scanning microscope. The inset shows an enlargement of the boxed area. Scale bar, 10 µm.
(6.50 MB TIF)

Figure S5 The effect of trypsin on the internalization of DiI-labeled virions. Labeled Ebola VLPs were adsorbed to Vero cells grown in 35 mm glass-bottom culture dishes for 30 min on ice. (A) The cells were treated with (middle and right panels) or without (left panel) 0.25% trypsin for 5 min at 37°C before (middle panel) and after (right panel) incubation for 2 h at 37°C followed by an additional incubation at 37°C for 1 h. The internalization of DiI-virions was analyzed by using confocal laser scanning microscope. Outlines of individual cells were drawn. Scale bars, 10 µm. (B) The internalized DiI-virions were measured in 10 individual cells. Each experiment was performed in triplicate and the results are presented as the mean ± SD (lower panels).
(0.70 MB TIF)

Figure S6 Dex Mw 10K associates with macropinosomes but not with CCPs and caveolae. Vero cells expressing eGFP-SNX5 (A), CLCa-eGFP (B, left panel), or Cav1-eGFP (B, right panel) were incubated with 0.5 mg/ml Alexa Fluor 647-Dex Mw 10K for 10 min at 37°C. The co-localization of Alexa Fluor-Dex Mw 10K (purple) with eGFP-SNX5, CLCa-eGFP, or Cav1-eGFP was analyzed by using confocal laser scanning microscope. The inset shows an enlargement of the boxed area. Scale bars, 10 µm.
(3.31 MB TIF)

Figure S7 Endogenous SNX5 and Rab7 co-localize with Ebola VLPs. (A) Vero cells were incubated with Ebola VLPs for 30 min on ice. The cells were then incubated for 10 min at 37°C and subsequently fixed in 4% PBS-buffered paraformaldehyde. Endogenous SNX5 (green) and Ebola VLPs (red) were immunostained by using an anti-SNX5 goat polyclonal antibody (Abcam) and an anti-VP40 rabbit polyclonal antibody, as well as Alexa Fluor 488- and 594-labeled secondary antibodies, respectively. Scale bar, 10 µm. (B) Vero cells were incubated with Ebola VLPs for 30 min on ice. The cells were then incubated for 10 min at 37°C and subsequently fixed in 4% PBS-buffered paraformaldehyde. Endogenous Rab7 (green) and Ebola VLPs (red) were immunostained by using an anti-Rab7 mouse monoclonal antibody (Abcam) and an anti-VP40 rabbit polyclonal antibody, as well as Alexa Fluor 488- and 594-labeled secondary antibodies, respectively. Scale bar, 10 µm.
(1.21 MB TIF)

Figure S8 Internalized Dex Mw 10K co-localizes with Rab7-positive vesicles. Vero cells expressing eGFP-Rab7 were incubated with 0.5 mg/ml Alexa Fluor 647-Dex Mw 10K for 30 min at 37°C. The co-localization of internalized Dex Mw 10K (purple) with GFP-Rab7 was analyzed by using laser scanning confocal microscope. The inset shows an enlargement of the boxed area. Scale bar, 10 µm.
(2.21 MB EPS)

Figure S9 Effect of NH4Cl on internalized DiI-labeled EBOV virions. Vero cells expressing eGFP-Rab7 were pretreated with 20 mM NH4Cl for 30 min at 37°C (right panel), or left untreated (Control; left panel). DiI-EbolaΔVP30 virions (red) were adsorbed to Vero cells expressing eGFP-Rab7 for 30 min on ice in the presence or absence of NH4Cl. Cells were then incubated for 4 h at 37°C in the presence or absence of NH4Cl and the internalized DiI-EbolaΔVP30 virions were analyzed by using confocal laser scanning microscope. The insets show enlargements of the boxed areas. Scale bars, 10 µm.
(1.14 MB TIF)

Figure S10 DiI-Ebola VLPs possessing a fusion-deficient GP mutant (F535R) co-localized with eGFP-Rab7-positive vesicles but failed to fuse with the membrane of Rab7-positive vesicles. DiI-Ebola VLPs possessing GP mutant (F535R) [Ebola VLP (GP-F535R)] (red) were adsorbed to eGFP-Rab7-expressing Vero cells for 30 min on ice. The cells were then incubated for 4 h at 37°C and the co-localization of DiI-virions with eGFP-Rab7 was analyzed by using confocal laser scanning microscope. Insets show enlargements of the boxed areas. Scale bar, 10 µm.
(3.17 MB EPS)

Figure S11 Effect of macropinocytosis inhibitors on the uptake of Dex Mw 10K. (A) Vero cells were pretreated with 2 µM cytochalasin D (CytoD), 50 nM wortmannin (Wort), 50 µM LY294002 hydrochloride, or 100 µM EIPA for 30 min at 37°C. Vero cells were incubated with 0.5 mg/ml AlexaFluor 647-Dex Mw 10K for 60 min at 37°C in the presence of inhibitors, harvested by trypsin, washed twice with ice-cold PBS and fixed with 4% PBS-buffered paraformaldehyde for 10 min at room temperature. As a control, Vero cells were treated with DMSO. The mean fluorescence intensities in the cells were analyzed by using flow cytometry. Each experiment was performed in triplicate and the mean fluorescence intensity is presented as the mean ± SD. (B) Representative images are shown. Outlines of individual cells are drawn. Scale bar, 10 µm.
(17.87 MB TIF)

Figure S12 Effect of macropinocytosis inhibitors on the co-localization of DiI-labeled influenza viruses with Rab7-positive vesicles. Vero cells expressing eGFP-Rab7 were pretreated with cytochalasin D (CytoD), wortmannin (Wort), LY294002, or EIPA for 30 min at 37°C. DiI-influenza viruses (red) were adsorbed to the cells for 30 min on ice, then incubated at 37°C for 2 h in the presence of inhibitors. As a control, DMSO-treated cells were incubated with DiI-influenza viruses (Control). Representative images acquired 2 h after the temperature shift are shown. DiI-influenza virions that co-localized with eGFP-Rab7-positive vesicles are indicated by arrows. Scale bars, 10 µm.
(1.73 MB TIF)

Figure S13 The effect of the internalization of DiI-labeled Ebola VLPs on dextran uptake. Vero cells, grown on cover slips, were incubated with 0.5 mg/ml Alexa Fluor 647-Dex Mw 10K in the absence or presence of Ebola VLPs for 60 min at 37°C. The uptake of Alexa Fluor 647-Dex Mw 10K was analyzed by using confocal laser scanning microscope. The effect of EIPA pretreatment was assessed in parallel. Scale bars, 10 µm.
(0.82 MB TIF)

Figure S14 The effect of PKC, Cdc 42, and Pak1 on the internalization of Ebola VLPs and Dex Mw 10K. (A) Effect of PKC inhibitors on the internalization of DiI-labeled Ebola virions and Dex 10K. Vero cells were treated with DMSO or staurosporine (Stauro) for 30 min at 37°C. Labeled Ebola VLPs were adsorbed to the cells for 30 min on ice and incubated for 2 h at 37°C in the absence or presence of inhibitor. Alexa Fluor-Dex Mw 10K was incubated for 2 h at 37°C in the absence or presence of inhibitor. Surface-bound virions or Dex Mw 10K were removed by trypsin and the internalization of DiI-virions (left panels) or Dex Mw 10K (right panels) was analyzed by using confocal laser scanning microscope. Outlines of individual cells are drawn. Scale bars, 10 µm. (B) Effect of down-regulation of Cdc42 and Pak1 on the internalization of DiI-labeled Ebola virions and Dex Mw 10K. Vero cells were transfected with control (Cont) non-targeting siRNA or siRNA to down-regulate Cdc42 and Pak1 expression. Labeled Ebola VLPs were adsorbed to the siRNA-transfected cells for 30 min on ice, 48 h post-transfection, and incubated for 2 h at 37°C. Alexa Fluor-Dex Mw 10K was incubated for 2 h at 37°C, 48 h post-transfection. After incubation for 2 h at 37°C, surface-bound virions or Dex Mw 10K were removed by trypsin for 5 min at 37°C. The internalization of DiI-virions (left panels) or Dex Mw 10K (right panels) was analyzed by using confocal laser scanning microscope. Outlines of individual cells are drawn. Scale bars, 10 µm.
(1.12 MB TIF)

Figure S15 Significant membrane ruffling was not observed in the absence of EBOV virions. eGFP-actin-expressing Vero cells were placed on ice for 30 min. The cells were then incubated at 37°C and time-lapse images were acquired at 15-second intervals over a 10 min time period by using a confocal laser scanning microscope. Still frames at the indicated times (sec) after the temperature shift to 37°C are shown. Scale bar, 10 µm.
(0.93 MB TIF)

Figure S16 DiI-labeled VSV pseudotyped with EBOV GP did not co-localize with CLCa-eGFP and Cav1-eGFP. DiI-VSV pseudotyped with EBOV GP (VSV*G-GP) (red) were adsorbed to CLCa-eGFP- or Cav1-eGFP-expressing Vero cells for 30 min on ice. The cells were then incubated for 10 min at 37°C and the co-localization of internalized DiI-virions with CLCa-eGFP or Cav1-eGFP was analyzed by use of confocal laser scanning microscope. Scale bars, 10 µm.
(5.80 MB TIF)

Figure S17 DiI-labeled VSV pseudotyped with VSV-G co-localized with CCPs. DiI-VSV pseudotyped with VSV-G (VSV*G-G) virions (red) were adsorbed to CLCa-eGFP-expressing Vero cells for 30 min on ice. The cells were then incubated for 10 min at 37°C and the co-localization of internalized DiI-pseudovirions with CLCa-eGFP was analyzed by use of confocal laser scanning microscope. DiI-pseudovirions that co-localized with CLCa-eGFP are indicated by arrows. Scale bar, 10 µm.
(4.30 MB TIF)

Table S1 Summary of siRNA target sequence and oligonucleotide sequence for RT-PCR
(0.03 MB DOC)

Text S1 Supporting materials and methods
(0.03 MB DOC)

Video S1 DiI-labeled Ebola-VLPs were efficiently internalized into cells after a temperature shift. DiI-labeled Ebola VLPs were adsorbed to Vero cells for 30 min on ice. Cells were then incubated for 15 min at 37°C and images were collected every 20 seconds by confocal laser scanning microscope. Ebola VLPs (red) were internalized immediately after the temperature shift.
(0.11 MB AVI)

Video S2 DiI-labeled Ebola-VLPs lacking GP were not internalized into cells after a temperature shift. DiI-labeled Ebola VLPs that lacked EBOV GP were adsorbed to Vero cells for 30 min on ice. Cells were then incubated for 15 min at 37°C and images were collected every 20 seconds by confocal laser scanning microscope. Ebola VLPs (red) remained stationary after the temperature shift.
(0.02 MB MOV)

Video S3 DiI-labeled Ebola-VLPs were not associated with CLCa-eGFP. DiI-labeled EbolaΔVP30 virions were adsorbed to Vero cells expressing CLCa-eGFP for 30 min on ice. Cells were then incubated for 15 min at 37°C and images were collected every 20 seconds by confocal laser scanning microscope. Co-localization of CLCa-eGFP (green) with DiI-virions (red) was not observed.
(5.38 MB AVI)

Video S4 DiI-labeled Ebola-VLPs were not associated with Cav1-eGFP. DiI-labeled EbolaΔVP30 virions were adsorbed to Vero cells expressing Cav1-eGFP for 30 min on ice. Cells were then incubated for 15 min at 37°C and images were collected every 20 seconds by confocal laser scanning microscope. Co-localization of Cav1-eGFP (green) with DiI-virions (red) was not observed.
(0.35 MB AVI)

Video S5 Internalized DiI-labeled EbolaΔVP30 virions were co-localized with eGFP-SNX5. DiI-labeled EbolaΔVP30 virions were adsorbed to Vero cells expressing eGFP-SNX5 for 30 min on ice. Cells were then incubated for 15 min at 37°C and images were collected every 20 seconds by confocal laser scanning microscope. DiI-EbolaΔVP30 virions (red) co-localized with eGFP-SNX5 (green).
(0.43 MB AVI)

Video S6 Internalized DiI-labeled influenza virus virions were not co-localized with eGFP-SNX5. DiI-labeled influenza virions were adsorbed to Vero cells expressing eGFP-SNX5 for 30 min on ice. Cells were then incubated for 15 min at 37°C and images were collected every 20 seconds by confocal laser scanning microscope. Co-localization of eGFP-SNX5 (green) with DiI-influenza virions (red) was not observed.
(0.32 MB AVI)

Video S7 Internalization of DiI-labeled EbolaΔVP30 virions was associated with plasma membrane ruffling. DiI-labeled EbolaΔVP30 virions were adsorbed to Vero cells expressing eGFP-actin for 30 min on ice. Cells were then incubated for 10 min at 37°C and images were collected every 10 seconds by confocal laser scanning microscope. Internalization of DiI-EbolaΔVP30 virions (red) was associated with plasma membrane ruffling (green).
(0.24 MB AVI)

Video S8 Plasma membrane ruffling was not observed in the absence of Ebola virions. Vero cells expressing eGFP-actin was placed on ice for 30 min. Cells were then incubated for 10 min at 37°C and images were collected every 10 seconds by confocal laser scanning microscope. Plasma membrane ruffling was not observed in this condition.
(0.44 MB AVI)
