# Supplementary figures and images for: Ebolavirus Is Internalized into Host Cells via Macropinocytosis in a Viral Glycoprotein-Dependent Manner
Source: PLoS Pathog. 2010 Sep 23;6(9):e1001121. doi: 10.1371/journal.ppat.1001121 (PMC2944813; doi:10.1371/journal.ppat.1001121)

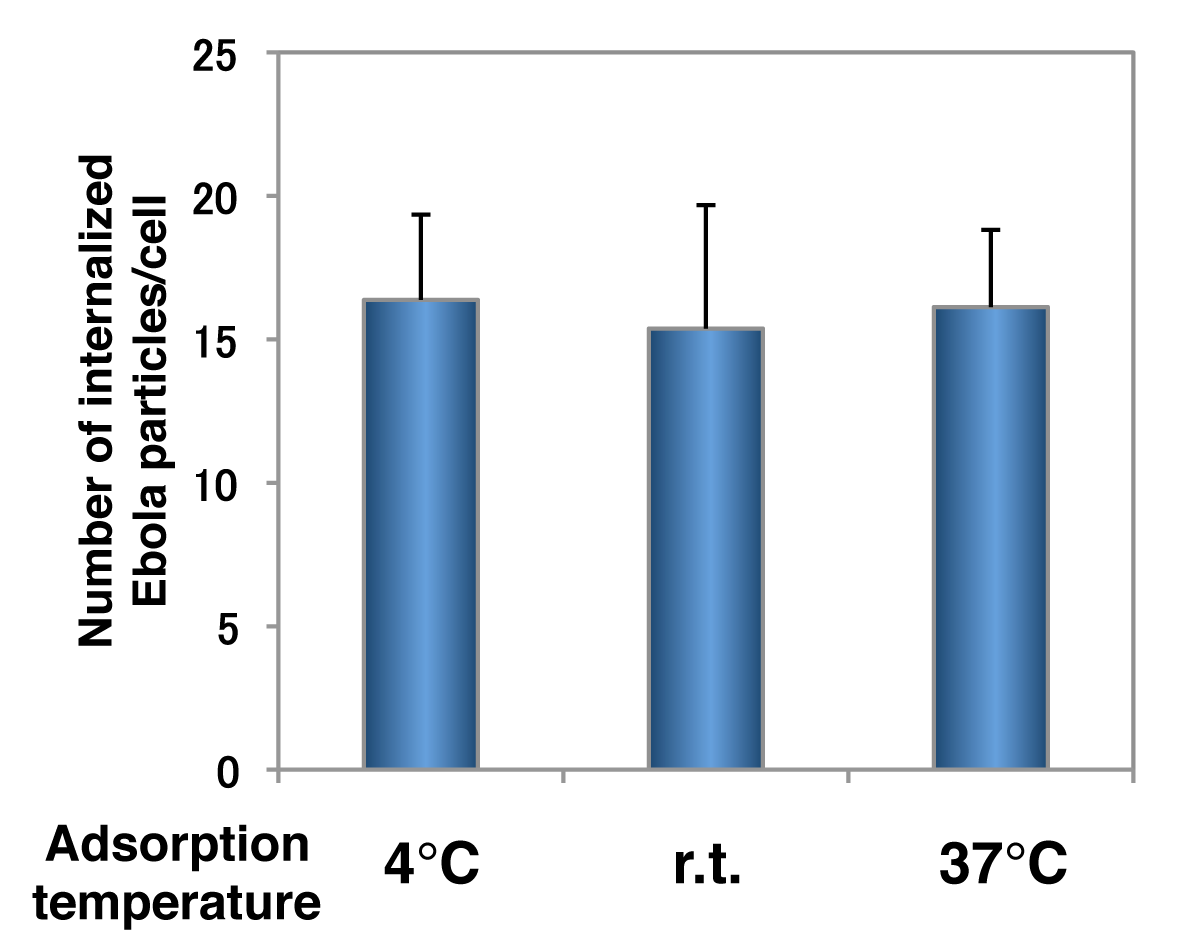

Supplement: Figure S1 — The effect of adsorption temperature on Ebola virion internalization. DiI-labeled Ebola VLPs were adsorbed to Vero cells grown in 35 mm glass-bottom culture dishes for 30 min on ice (4°C), room temperature (r.t.), or 37°C in parallel. The cells were then incubated for 2 h at 37°C. Surface-bound virions were removed by trypsin and the internalization of the DiI-virions was measured in 10 individual cells by use of confocal laser scanning microscope. Each experiment was performed in triplicate and the results are presented as the mean ± SD. (3.40 MB TIF) [file ppat.1001121.s001.tif]

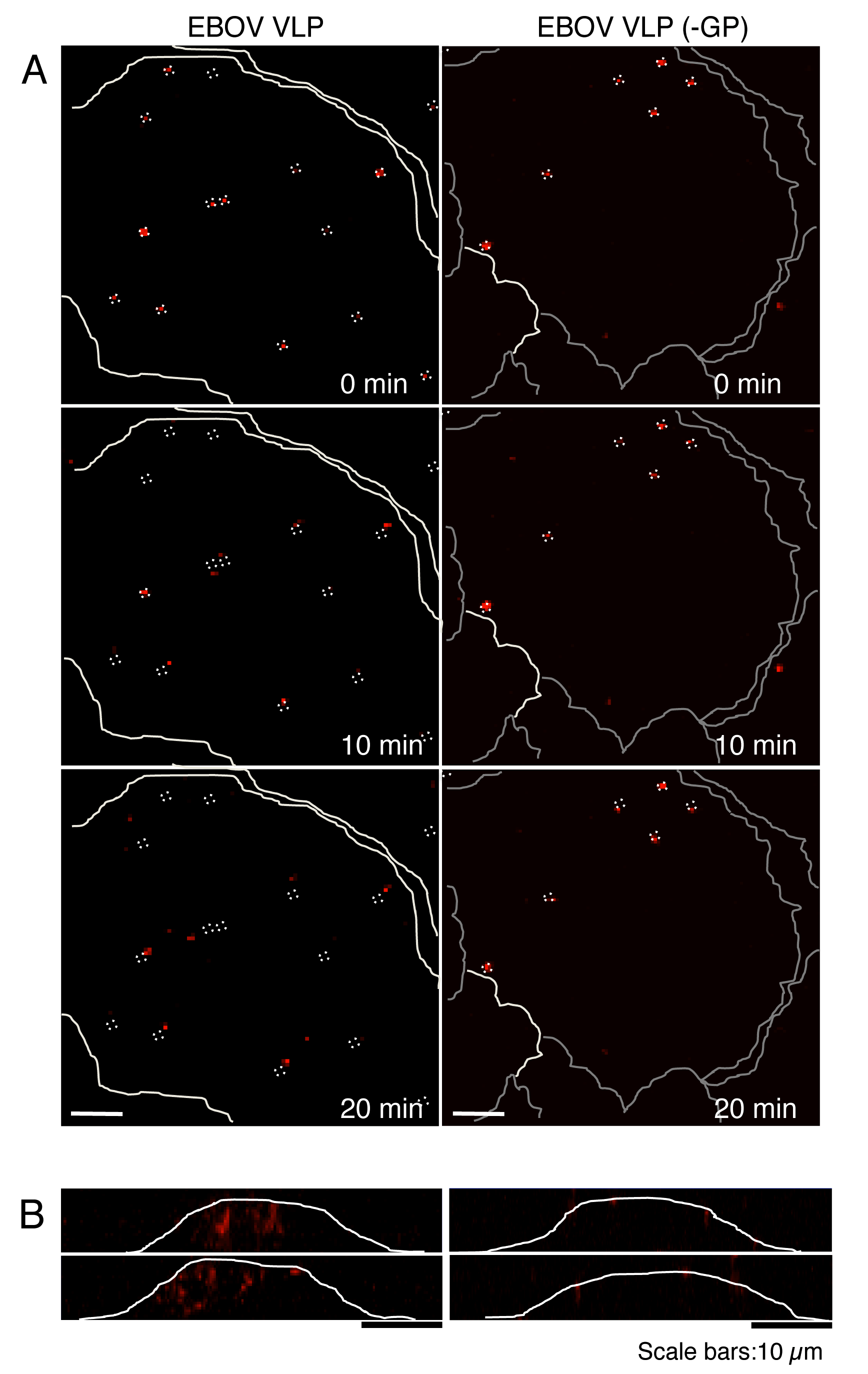

Supplement: Figure S2 — Visualization of the internalization of DiI-labeled EBOV particles in live cells. (A) DiI-labeled Ebola-VLPs (red; left panel) or control VLPs lacking GP [(Ebola VLPs (-GP)] (red; right panel) were absorbed to Vero cells for 30 min on ice. The cells were incubated at 37°C and time-lapse images were acquired at 20-second intervals over a period of 20 min by using a confocal laser scanning microscope. Still frames at the indicated times (min) after the temperature shift to 37°C are shown. Individual cells are highlighted. Initial positions of individual viral particles are shown as white dots. Scale bars, 10 µm. (B) DiI-labeled Ebola VLPs (red; left panel) or Ebola VLPs (-GP) (red; right panel) were absorbed to Vero cells for 30 min on ice. The cells were then incubated for 30 min at 37°C. Images were collected by taking 10∼15 optical slices of z-stack in 0.16 µm steps and the cross-sectional views were processed with LSM510 software. Outlines of individual cells were drawn. Scale bars, 10 µm. (1.04 MB TIF) [file ppat.1001121.s002.tif]

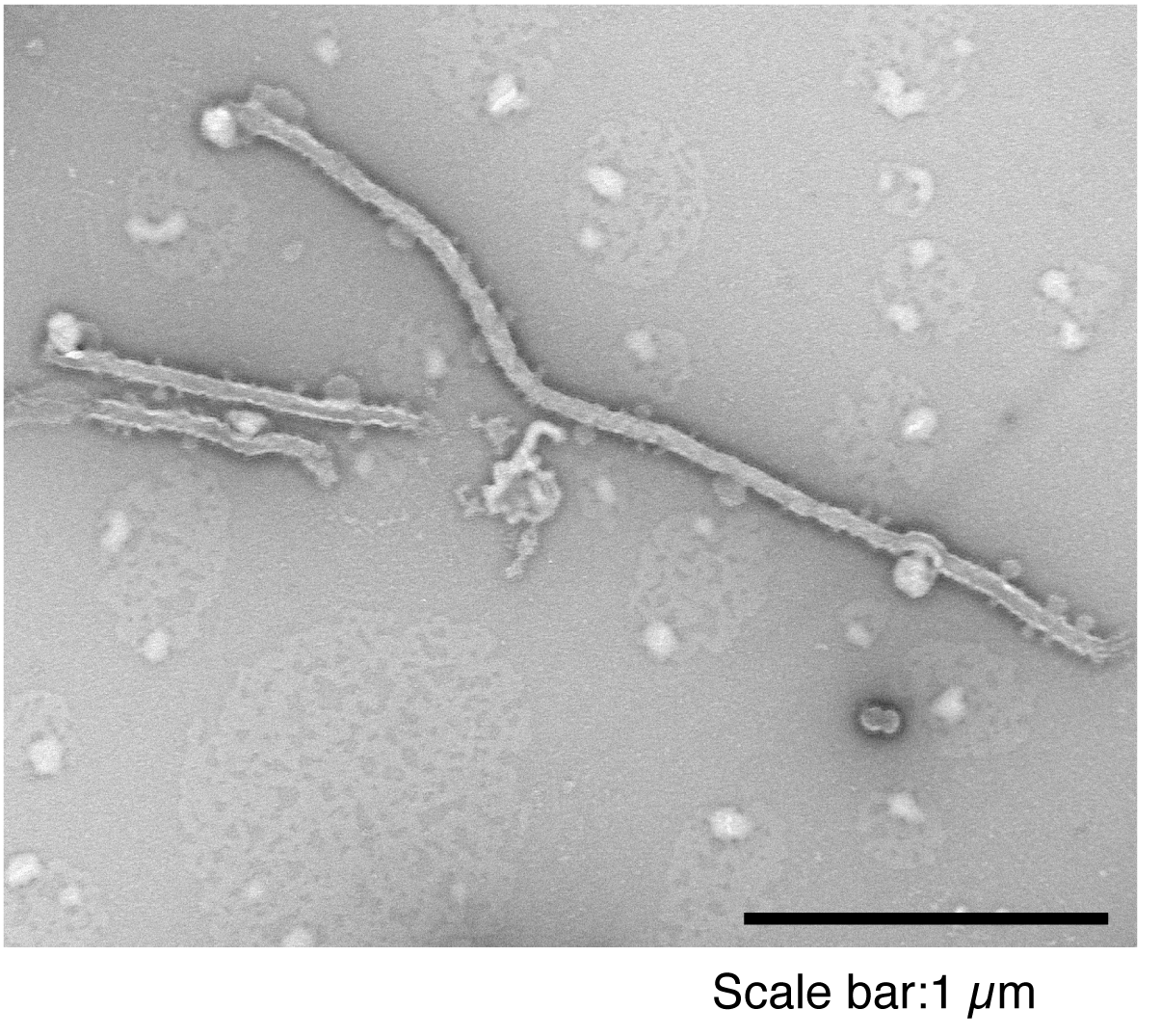

Supplement: Figure S3 — Filamentous morphologies of Ebola VLPs. Ebola VLPs released into the supernatants of 293T cells expressing EBOV VP40, NP and GP were purified as described in the Materials and Methods and then negatively stained with 1% uranyl acetate. Filamentous particles of various lengths with surface spikes can be seen. Scale bar, 1 µm. (3.91 MB TIF) [file ppat.1001121.s003.tif]

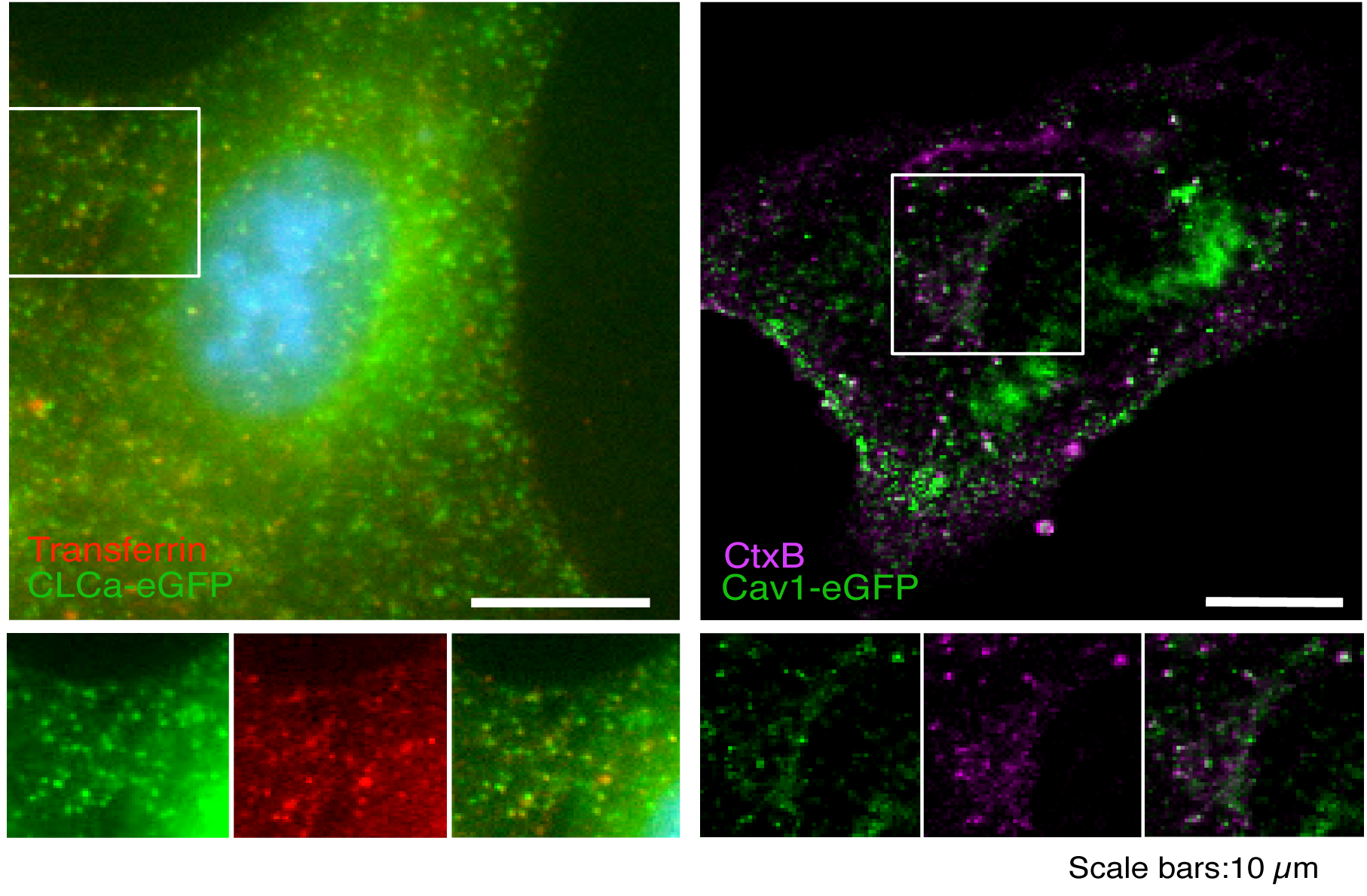

Supplement: Figure S4 — Transferrin and cholera toxin subunit B are co-localized with CLCa-eGFP and Cav1-eGFP, respectively. (Left panel) Vero cells expressing CLCa-eGFP were incubated with 2 µg/ml Alexa Fluor 594-Transferrin (Tf) (red) for 30 min on ice. The cells were then incubated for 3 min at 37°C and subsequently fixed in PBS-buffered 4% paraformaldehyde. The co-localization of Alexa Fluor-Tf with CLCa-eGFP was analyzed by using confocal laser scanning microscope. The inset shows an enlargement of the boxed area. Scale bar, 1 µm. (Right panel) Vero cells expressing Cav1-eGFP were incubated with 2 µg/ml Alexa Fluor 647-cholera toxin subunit B (CtxB) (purple) for 30 min on ice. The cells were then incubated for 60 min at 37°C and subsequently fixed in PBS-buffered 4% paraformaldehyde. The co-localization of Alexa Fluor-CtxB with Cav1-eGFP was analyzed by use of a confocal laser scanning microscope. The inset shows an enlargement of the boxed area. Scale bar, 10 µm. (6.50 MB TIF) [file ppat.1001121.s004.tif]

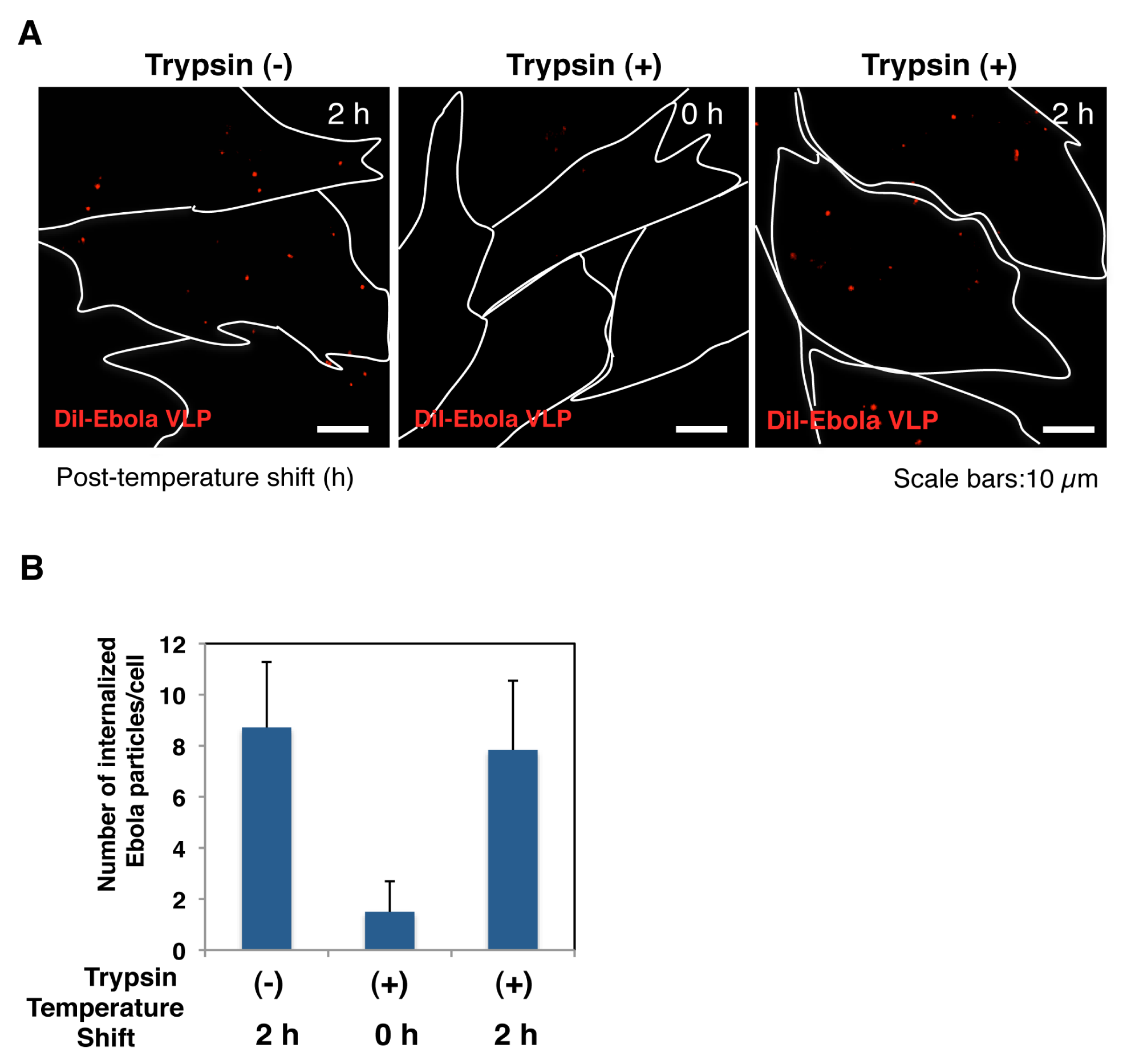

Supplement: Figure S5 — The effect of trypsin on the internalization of DiI-labeled virions. Labeled Ebola VLPs were adsorbed to Vero cells grown in 35 mm glass-bottom culture dishes for 30 min on ice. (A) The cells were treated with (middle and right panels) or without (left panel) 0.25% trypsin for 5 min at 37°C before (middle panel) and after (right panel) incubation for 2 h at 37°C followed by an additional incubation at 37°C for 1 h. The internalization of DiI-virions was analyzed by using confocal laser scanning microscope. Outlines of individual cells were drawn. Scale bars, 10 µm. (B) The internalized DiI-virions were measured in 10 individual cells. Each experiment was performed in triplicate and the results are presented as the mean ± SD (lower panels). (0.70 MB TIF) [file ppat.1001121.s005.tif]

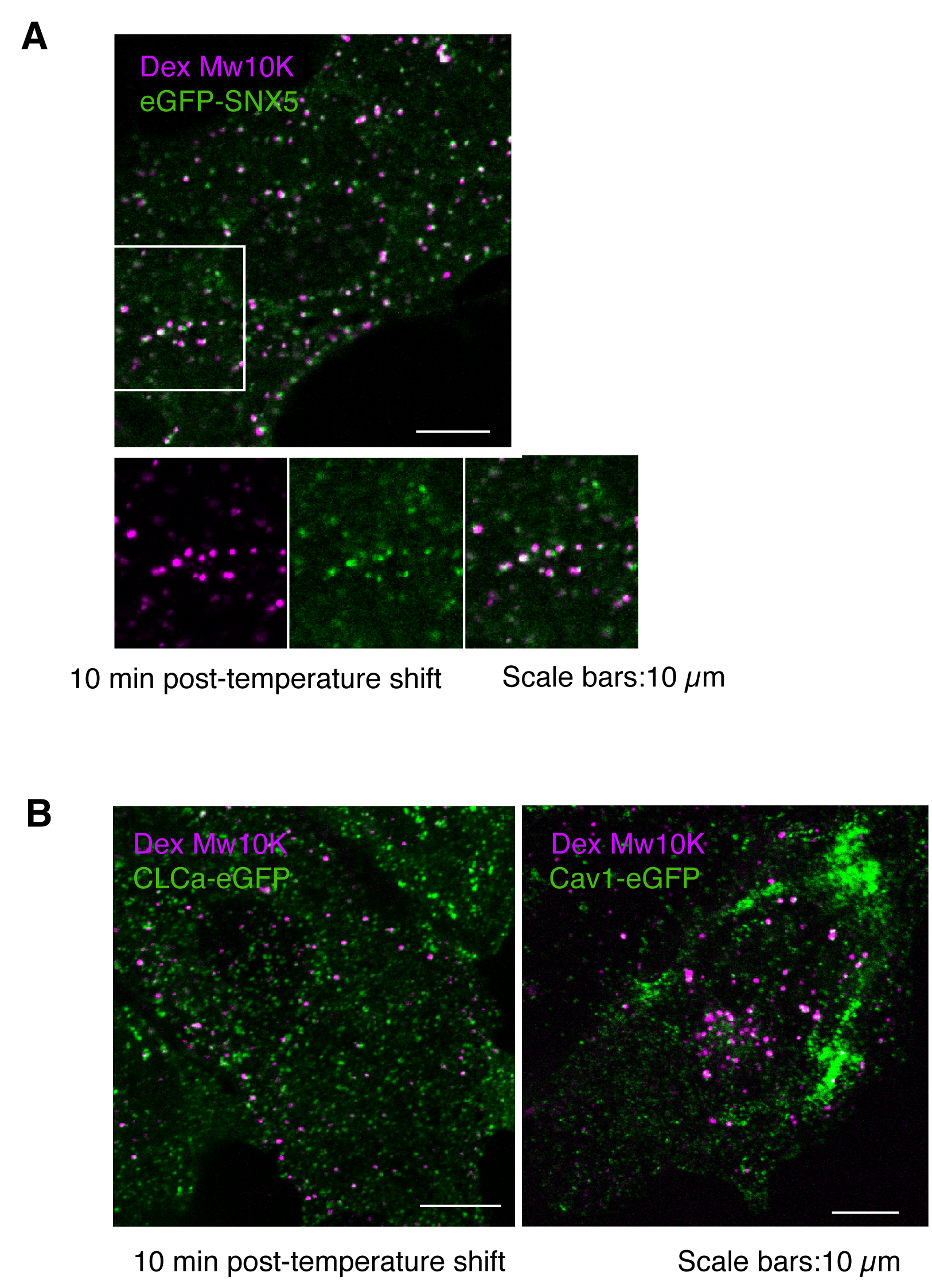

Supplement: Figure S6 — Dex Mw 10K associates with macropinosomes but not with CCPs and caveolae. Vero cells expressing eGFP-SNX5 (A), CLCa-eGFP (B, left panel), or Cav1-eGFP (B, right panel) were incubated with 0.5 mg/ml Alexa Fluor 647-Dex Mw 10K for 10 min at 37°C. The co-localization of Alexa Fluor-Dex Mw 10K (purple) with eGFP-SNX5, CLCa-eGFP, or Cav1-eGFP was analyzed by using confocal laser scanning microscope. The inset shows an enlargement of the boxed area. Scale bars, 10 µm. (3.31 MB TIF) [file ppat.1001121.s006.tif]

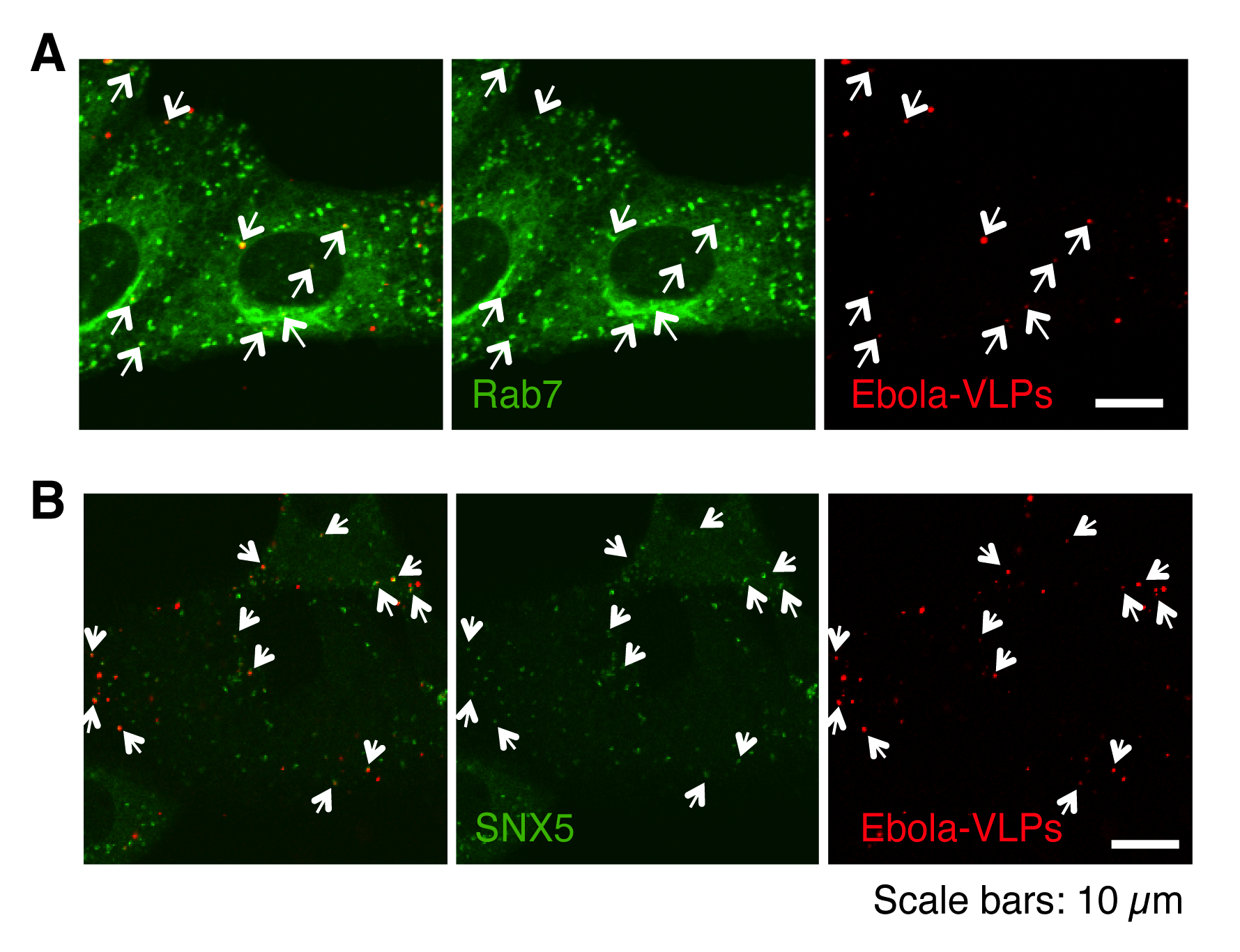

Supplement: Figure S7 — Endogenous SNX5 and Rab7 co-localize with Ebola VLPs. (A) Vero cells were incubated with Ebola VLPs for 30 min on ice. The cells were then incubated for 10 min at 37°C and subsequently fixed in 4% PBS-buffered paraformaldehyde. Endogenous SNX5 (green) and Ebola VLPs (red) were immunostained by using an anti-SNX5 goat polyclonal antibody (Abcam) and an anti-VP40 rabbit polyclonal antibody, as well as Alexa Fluor 488- and 594-labeled secondary antibodies, respectively. Scale bar, 10 µm. (B) Vero cells were incubated with Ebola VLPs for 30 min on ice. The cells were then incubated for 10 min at 37°C and subsequently fixed in 4% PBS-buffered paraformaldehyde. Endogenous Rab7 (green) and Ebola VLPs (red) were immunostained by using an anti-Rab7 mouse monoclonal antibody (Abcam) and an anti-VP40 rabbit polyclonal antibody, as well as Alexa Fluor 488- and 594-labeled secondary antibodies, respectively. Scale bar, 10 µm. (1.21 MB TIF) [file ppat.1001121.s007.tif]

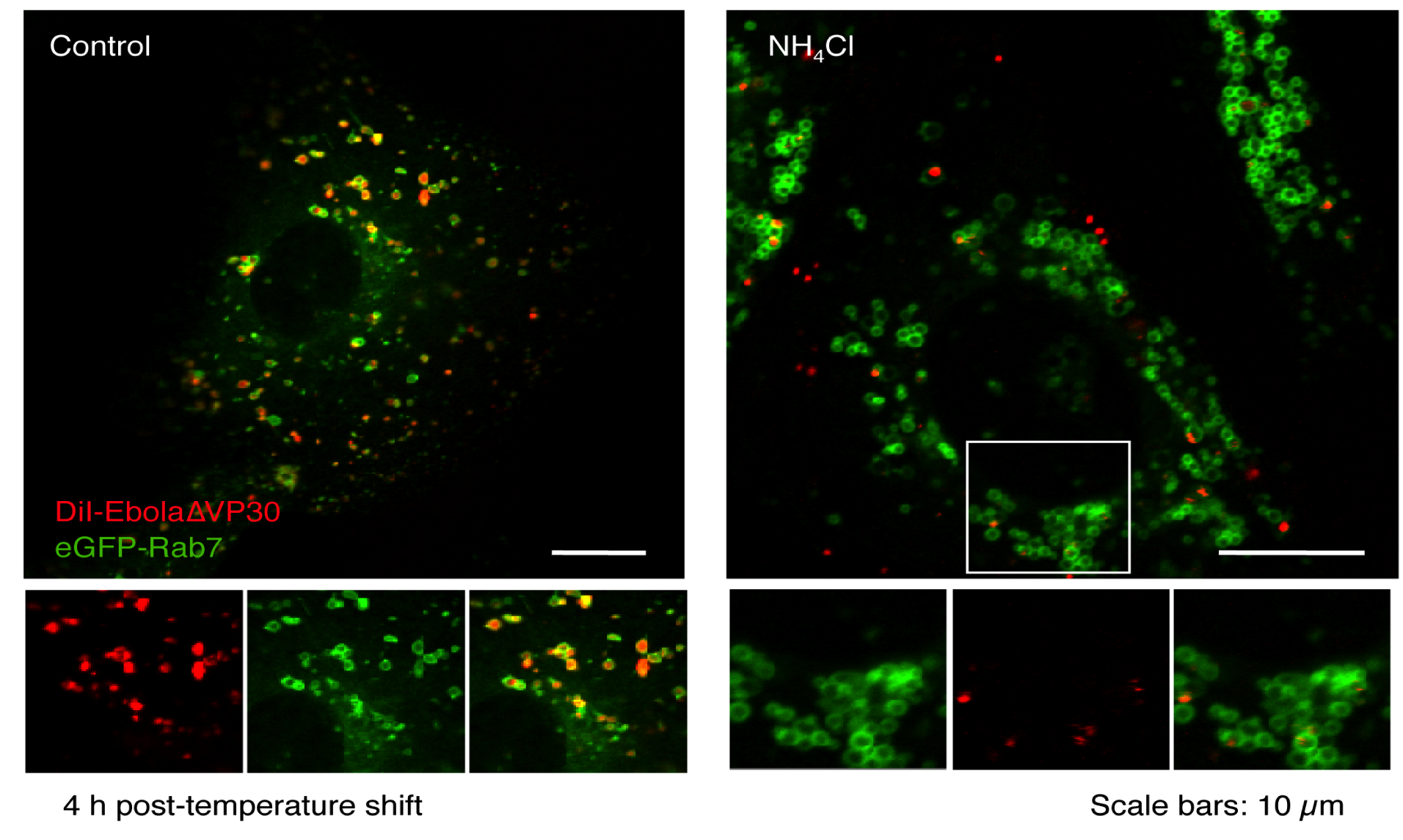

Supplement: Figure S9 — Effect of NH4Cl on internalized DiI-labeled EBOV virions. Vero cells expressing eGFP-Rab7 were pretreated with 20 mM NH4Cl for 30 min at 37°C (right panel), or left untreated (Control; left panel). DiI-EbolaΔVP30 virions (red) were adsorbed to Vero cells expressing eGFP-Rab7 for 30 min on ice in the presence or absence of NH4Cl. Cells were then incubated for 4 h at 37°C in the presence or absence of NH4Cl and the internalized DiI-EbolaΔVP30 virions were analyzed by using confocal laser scanning microscope. The insets show enlargements of the boxed areas. Scale bars, 10 µm. (1.14 MB TIF) [file ppat.1001121.s009.tif]

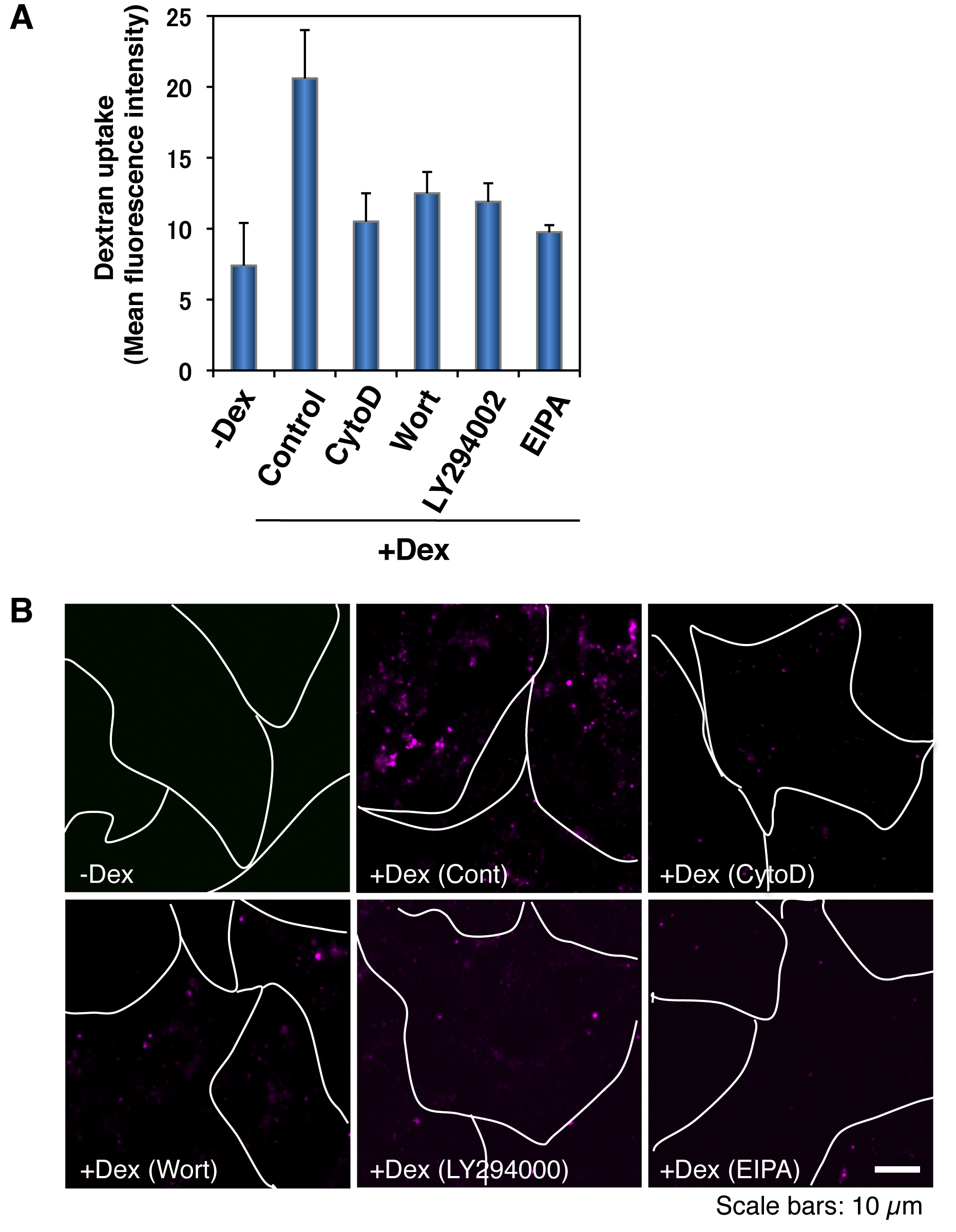

Supplement: Figure S11 — Effect of macropinocytosis inhibitors on the uptake of Dex Mw 10K. (A) Vero cells were pretreated with 2 µM cytochalasin D (CytoD), 50 nM wortmannin (Wort), 50 µM LY294002 hydrochloride, or 100 µM EIPA for 30 min at 37°C. Vero cells were incubated with 0.5 mg/ml AlexaFluor 647-Dex Mw 10K for 60 min at 37°C in the presence of inhibitors, harvested by trypsin, washed twice with ice-cold PBS and fixed with 4% PBS-buffered paraformaldehyde for 10 min at room temperature. As a control, Vero cells were treated with DMSO. The mean fluorescence intensities in the cells were analyzed by using flow cytometry. Each experiment was performed in triplicate and the mean fluorescence intensity is presented as the mean ± SD. (B) Representative images are shown. Outlines of individual cells are drawn. Scale bar, 10 µm. (17.87 MB TIF) [file ppat.1001121.s011.tif]

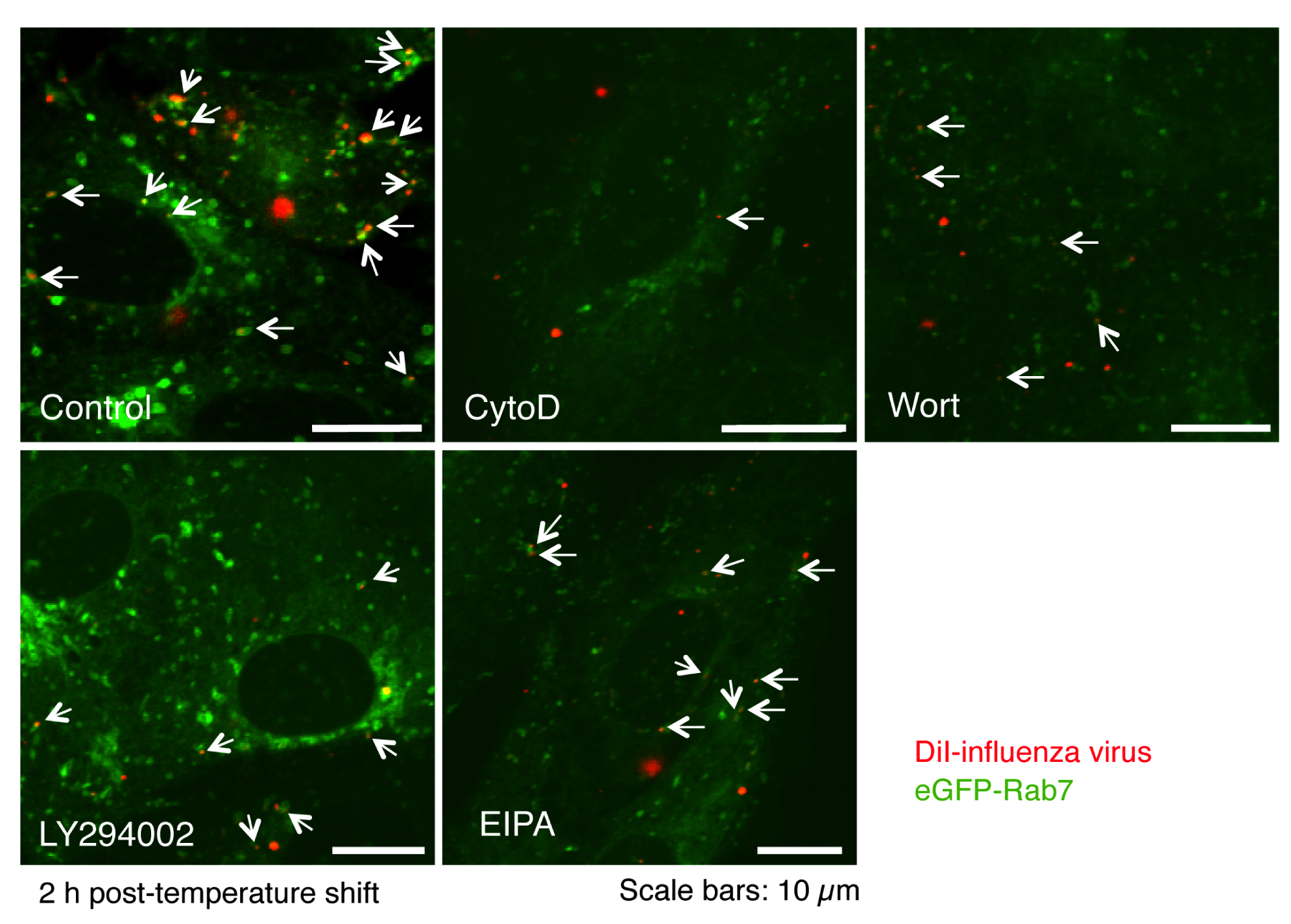

Supplement: Figure S12 — Effect of macropinocytosis inhibitors on the co-localization of DiI-labeled influenza viruses with Rab7-positive vesicles. Vero cells expressing eGFP-Rab7 were pretreated with cytochalasin D (CytoD), wortmannin (Wort), LY294002, or EIPA for 30 min at 37°C. DiI-influenza viruses (red) were adsorbed to the cells for 30 min on ice, then incubated at 37°C for 2 h in the presence of inhibitors. As a control, DMSO-treated cells were incubated with DiI-influenza viruses (Control). Representative images acquired 2 h after the temperature shift are shown. DiI-influenza virions that co-localized with eGFP-Rab7-positive vesicles are indicated by arrows. Scale bars, 10 µm. (1.73 MB TIF) [file ppat.1001121.s012.tif]

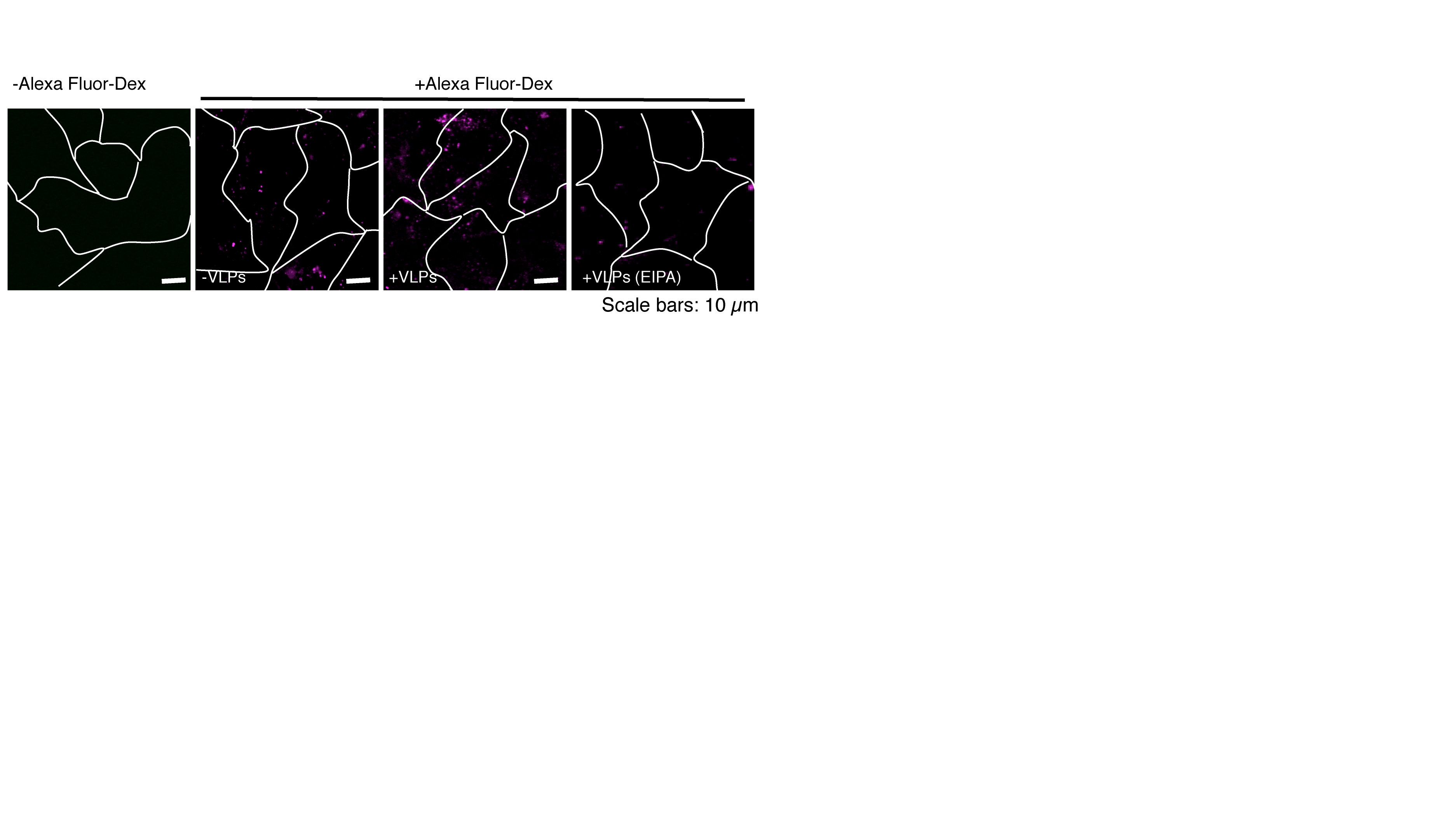

Supplement: Figure S13 — The effect of the internalization of DiI-labeled Ebola VLPs on dextran uptake. Vero cells, grown on cover slips, were incubated with 0.5 mg/ml Alexa Fluor 647-Dex Mw 10K in the absence or presence of Ebola VLPs for 60 min at 37°C. The uptake of Alexa Fluor 647-Dex Mw 10K was analyzed by using confocal laser scanning microscope. The effect of EIPA pretreatment was assessed in parallel. Scale bars, 10 µm. (0.82 MB TIF) [file ppat.1001121.s013.tif]

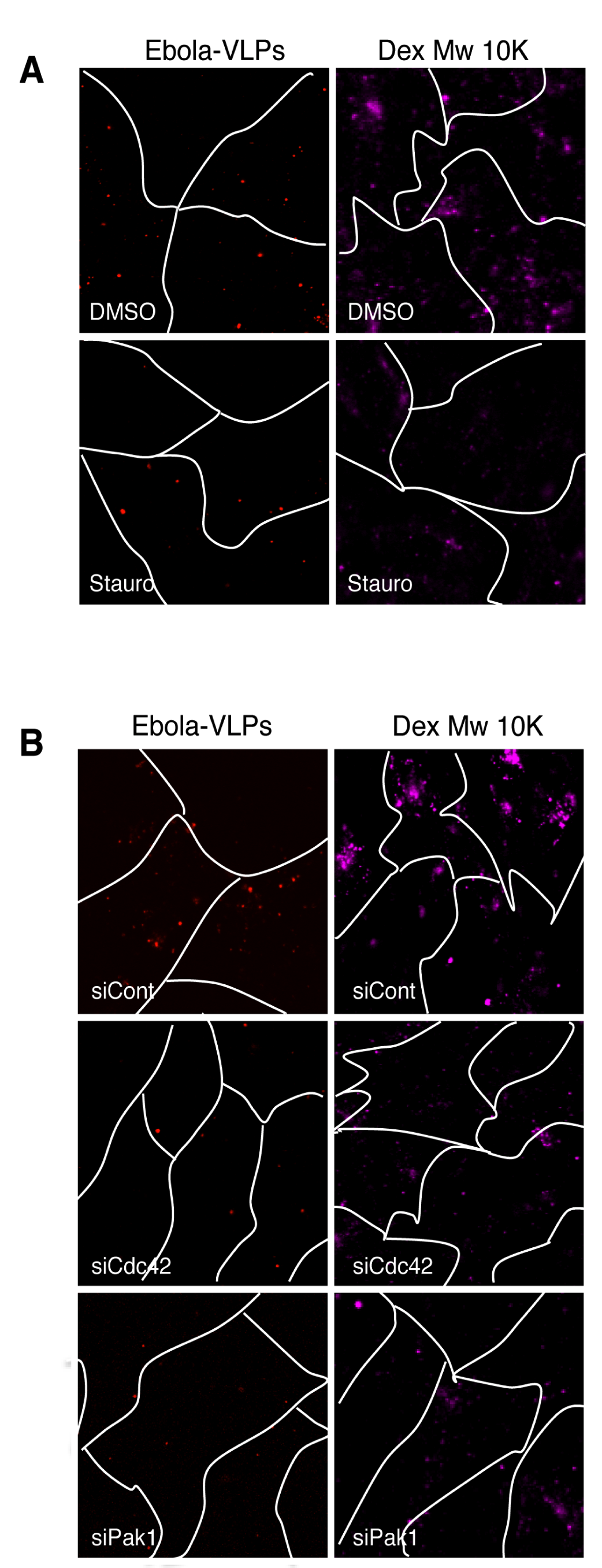

Supplement: Figure S14 — The effect of PKC, Cdc 42, and Pak1 on the internalization of Ebola VLPs and Dex Mw 10K. (A) Effect of PKC inhibitors on the internalization of DiI-labeled Ebola virions and Dex 10K. Vero cells were treated with DMSO or staurosporine (Stauro) for 30 min at 37°C. Labeled Ebola VLPs were adsorbed to the cells for 30 min on ice and incubated for 2 h at 37°C in the absence or presence of inhibitor. Alexa Fluor-Dex Mw 10K was incubated for 2 h at 37°C in the absence or presence of inhibitor. Surface-bound virions or Dex Mw 10K were removed by trypsin and the internalization of DiI-virions (left panels) or Dex Mw 10K (right panels) was analyzed by using confocal laser scanning microscope. Outlines of individual cells are drawn. Scale bars, 10 µm. (B) Effect of down-regulation of Cdc42 and Pak1 on the internalization of DiI-labeled Ebola virions and Dex Mw 10K. Vero cells were transfected with control (Cont) non-targeting siRNA or siRNA to down-regulate Cdc42 and Pak1 expression. Labeled Ebola VLPs were adsorbed to the siRNA-transfected cells for 30 min on ice, 48 h post-transfection, and incubated for 2 h at 37°C. Alexa Fluor-Dex Mw 10K was incubated for 2 h at 37°C, 48 h post-transfection. After incubation for 2 h at 37°C, surface-bound virions or Dex Mw 10K were removed by trypsin for 5 min at 37°C. The internalization of DiI-virions (left panels) or Dex Mw 10K (right panels) was analyzed by using confocal laser scanning microscope. Outlines of individual cells are drawn. Scale bars, 10 µm. (1.12 MB TIF) [file ppat.1001121.s014.tif]

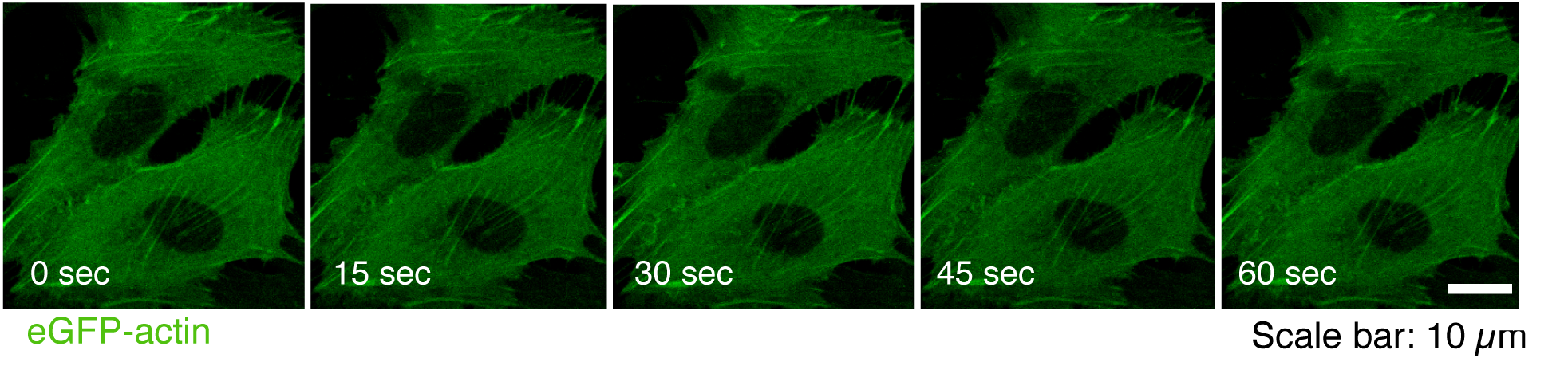

Supplement: Figure S15 — Significant membrane ruffling was not observed in the absence of EBOV virions. eGFP-actin-expressing Vero cells were placed on ice for 30 min. The cells were then incubated at 37°C and time-lapse images were acquired at 15-second intervals over a 10 min time period by using a confocal laser scanning microscope. Still frames at the indicated times (sec) after the temperature shift to 37°C are shown. Scale bar, 10 µm. (0.93 MB TIF) [file ppat.1001121.s015.tif]

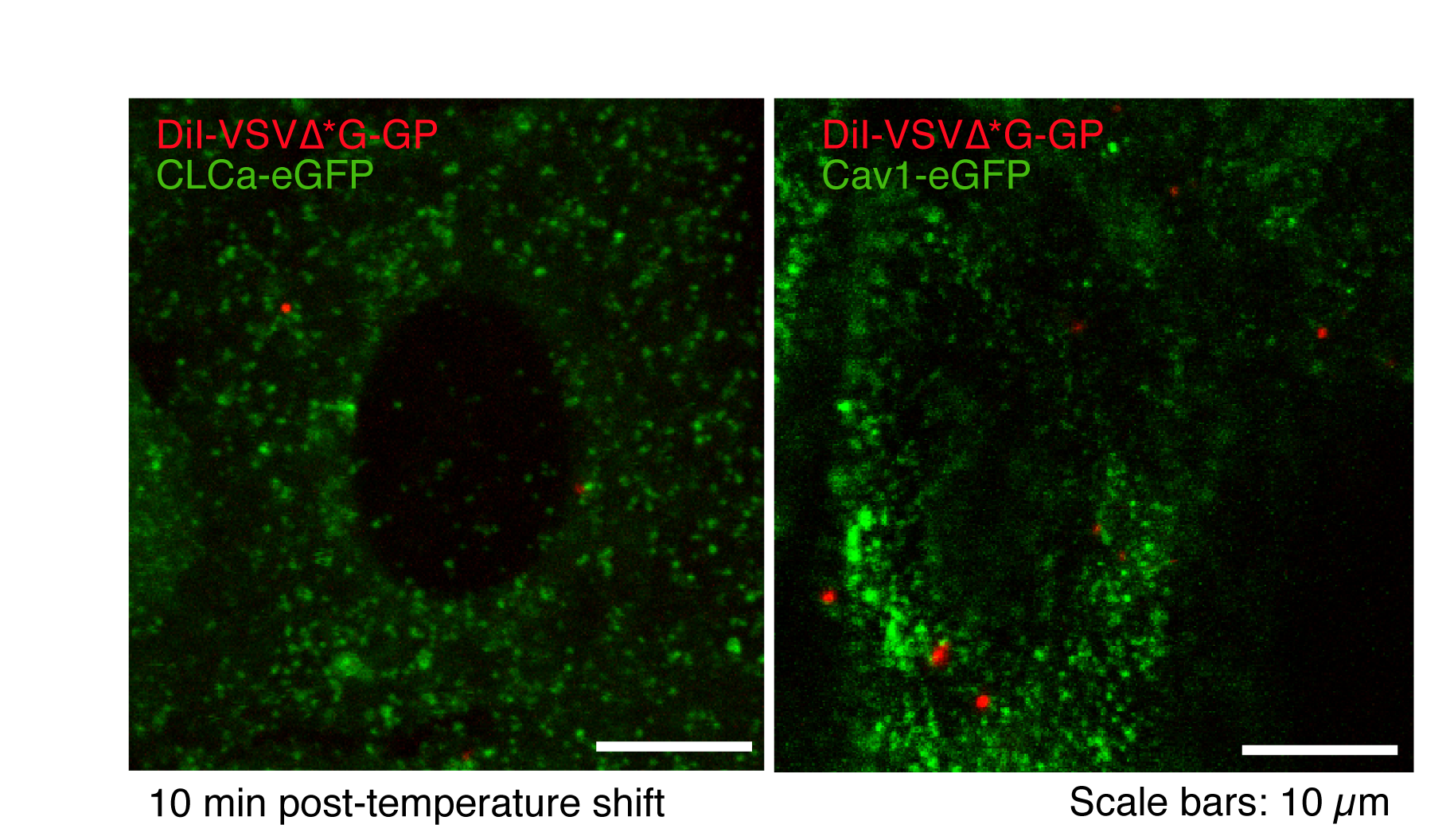

Supplement: Figure S16 — DiI-labeled VSV pseudotyped with EBOV GP did not co-localize with CLCa-eGFP and Cav1-eGFP. DiI-VSV pseudotyped with EBOV GP (VSV*G-GP) (red) were adsorbed to CLCa-eGFP- or Cav1-eGFP-expressing Vero cells for 30 min on ice. The cells were then incubated for 10 min at 37°C and the co-localization of internalized DiI-virions with CLCa-eGFP or Cav1-eGFP was analyzed by use of confocal laser scanning microscope. Scale bars, 10 µm. (5.80 MB TIF) [file ppat.1001121.s016.tif]

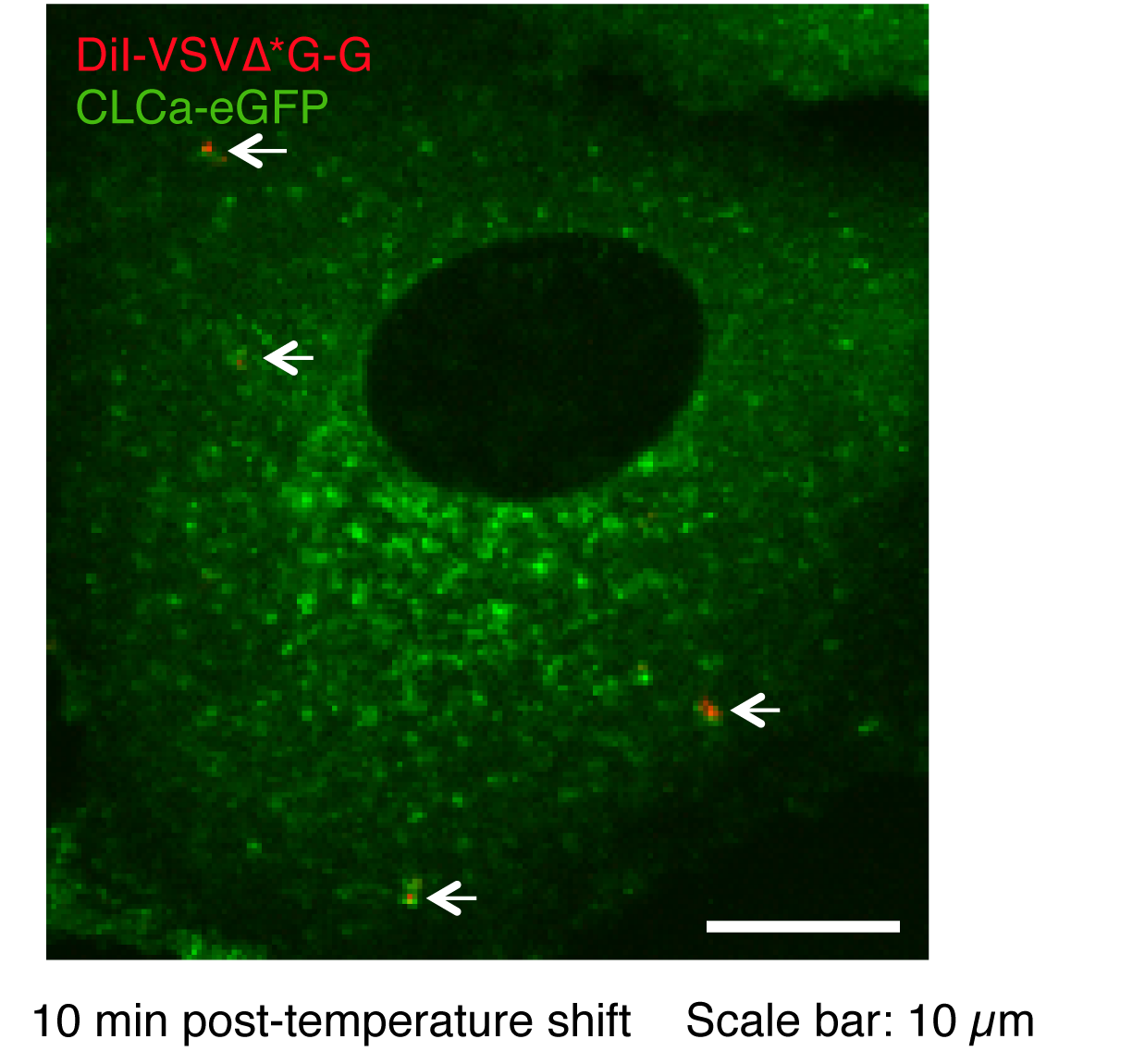

Supplement: Figure S17 — DiI-labeled VSV pseudotyped with VSV-G co-localized with CCPs. DiI-VSV pseudotyped with VSV-G (VSV*G-G) virions (red) were adsorbed to CLCa-eGFP-expressing Vero cells for 30 min on ice. The cells were then incubated for 10 min at 37°C and the co-localization of internalized DiI-pseudovirions with CLCa-eGFP was analyzed by use of confocal laser scanning microscope. DiI-pseudovirions that co-localized with CLCa-eGFP are indicated by arrows. Scale bar, 10 µm. (4.30 MB TIF) [file ppat.1001121.s017.tif]
