# Supplementary material for: Ebolavirus Is Internalized into Host Cells via Macropinocytosis in a Viral Glycoprotein-Dependent Manner
Source: PLoS Pathog. 2010 Sep 23;6(9):e1001121. doi: 10.1371/journal.ppat.1001121 (PMC2944813; doi:10.1371/journal.ppat.1001121)
Supplement: Table S1 — Summary of siRNA target sequence and oligonucleotide sequence for RT-PCR (0.03 MB DOC) [file ppat.1001121.s018.doc]

| **Cellular factors targeted by siRNA** | **Target sequences** | **Ref** | **Oligonucleotide sequences used for RT-PCR** |
| --- | --- | --- | --- |
| Clathrin heavy chain | AACAUUGGCUUCAGUACCCUG | 103 | Not applicable |
| Caveolin 1 | AAGATGTGATTGCAGAACCAG | 104 | Not applicable |
| Cdc42 | AAAGACTCCTTTCTTGCTTGT | 105 | Forward 5'-GCCCGTGACCTGAAGGCTGTCA-3'  Reverse 5'-TGCTTTTAGTATGATGCCGACACCA-3' |
| Pak1 | Purchased from Cell signaling Sequence not available | 106 | Forward5’AAAAGAACAGCAACATGACCTACGA’3  Reverse 5’CAGCAAATGGGTCGGGATC 3’ |

Supplementary Table 1. Summary of target sequences of siRNAs and oligonucleotide for RT-PCR used in this study
