# Supplementary material for: Ebolavirus Is Internalized into Host Cells via Macropinocytosis in a Viral Glycoprotein-Dependent Manner
Source: PLoS Pathog. 2010 Sep 23;6(9):e1001121. doi: 10.1371/journal.ppat.1001121 (PMC2944813; doi:10.1371/journal.ppat.1001121)
Supplement: Text S1 — Supporting materials and methods (0.03 MB DOC) [file ppat.1001121.s019.doc]

**Supporting materials and methods**

**Real-time analysis in live cells**

For real-time imaging of the internalization and the co-localization of DiI-labeled Ebola VLPs, VSV psedovirions, or influenza virus with CLCa-eGFP, Cav1-eGFP, or eGFP-Rab7, Vero cells or Vero cells expressing CLCa-eGFP, Cav1-eGFP, or eGFP-Rab7 were grown on 35 mm glass-bottom culture dishes. DiI-labeled viral particles were adsorbed to the cells for 30 min on ice, at room temperature, or 37°C in phenol red-free MEM (Invitrogen) containing 2% FBS and 4% BSA. The cells were washed with the same medium, then placed on a heated stage equipped to a confocal laser scanning microscope (LSM510 META, Carl Zeiss) and incubated at 37°C for various times. Time-lapse fluorescent images were acquired at 20-second intervals over a period of 30 min by using a confocal laser scanning microscope and were processed with LSM510 software.

**Negative staining**

Purified Ebola VLPs obtained from the culture media of 293T cells were mounted onto a Formvar-coated copper grid, stained with 1% uranyl acetate, and examined by use of an electron microscope (H-7650, HITACHI) at 100 kV.

**Transferrin and cholera toxin subunit B uptake assay**

Vero cells expressing CLCa-eGFP or Cav1-eGFP were incubated with 2 µg/ml AlexaFluor 594-Transferrin (Tf) (Invitrogen) or 2 µg/ml AlexaFluor 647-cholera toxin subunit B (CtxB) (Invitrogen) for 3 min or 1 h at 37°C, respectively, and subsequently fixed in 4% PBS-buffered paraformaldehyde. The co-localization of Alexa Fluor-Tf with CLCa-eGFP and Alexa Fluor-CtxB with Cav1-eGFP was analyzed by use of confocal laser scanning microscope.

**Ebola virion internalization analysis**

For analysis of the internalization of Ebola virions, DiI-Ebola VLPs were adsorbed to Vero cells grown in 35 mm glass-bottom culture dishes for 30 min on ice. The cells were treated with or without trypsin for 5 min at 37°C before and after incubation for 2 h at 37°C followed by an additional incubation for 1 h at 37°C. Images were acquired by using confocal laser scanning microscope and the internalized virions were measured in 10 individual cells. Each experiment was performed in triplicate and the results are presented as the mean ± SD.

**Fluid-phase uptake assay**

Vero cells expressing CLCa-eGFP, Cav1-eGFP, eGFP-SXN5 or eGFP-Rab7 were incubated with 0.5 mg/ml Alexa Fluor 647-Dex Mw 10K (Invitrogen) for 20 min at 37°C. Surface-bound Alexa Fluor-Dex Mw 10K was removed by washing the cells twice with ice-cold PBS, once with a low-pH wash buffer (0.1 M sodium acetate, 0.05 M NaCl, pH 5.5) for 10 min on ice, and then twice more with ice-cold PBS. Cells were then fixed with 10% PBS-buffered formamide for 10 min at room temperature. They were then washed with PBS twice and Dex Mw 10K uptake was analyzed by using confocal laser scanning microscope.

**Inhibitor treatment**

Vero cells or Vero cells expressing eGFP-Rab7 cultured in 35 mm glass-bottom culture dishes were pretreated with 20 mM NH_4_Cl, 2 µM cytochalasin D (Sigma-Aldrich), 50 nM wortmannin (Sigma-Aldrich), 50 µM LY294002 hydrochloride (Sigma-Aldrich), or 100 µM EIPA (Sigma-Aldrich) in phenol red-free MEM containing 2% FBS and 4% BSA for 30 min at 37°C. DiI-labeled virions were adsorbed to the cells for 30 min on ice in the presence of these inhibitors. Cells were then washed with the same medium and incubated for various times at 37°C in the presence of the inhibitors. As a control, the cells were treated with dimethyl sulfoxide (DMSO, Sigma-Aldrich). Efficiencies of co-localization of DiI-labeled viral particles with eGFP-Rab7 were determined by using confocal laser scanning microscope as described in the main text. To assess the effect of the inhibitors on fluid phase uptake, after pretreatment with the inhibitors, the Vero cells were incubated with 0.5 mg/ml Alexa Fluor 647-Dex Mw 10K, harvested by trypsin, washed twice with ice-cold PBS and fixed with 4% PBS-buffered paraformaldehyde for 10 min at room temperature. The mean fluorescence intensities in the cells were analyzed by use of flow cytometry (FACSCalibur; Becton Dickinson, Franklin Lakes, USA). Each experiment was performed in triplicate and the results are presented as the mean ± SD.
